# Improving polygenic risk prediction in admixed populations by explicitly modeling ancestral-differential effects via GAUDI

**DOI:** 10.1038/s41467-024-45135-z

**Published:** 2024-02-03

**Authors:** Quan Sun, Bryce T. Rowland, Jiawen Chen, Anna V. Mikhaylova, Christy Avery, Ulrike Peters, Jessica Lundin, Tara Matise, Steve Buyske, Ran Tao, Rasika A. Mathias, Alexander P. Reiner, Paul L. Auer, Nancy J. Cox, Charles Kooperberg, Timothy A. Thornton, Laura M. Raffield, Yun Li

**Affiliations:** 1 Department of Biostatistics, University of North Carolina at Chapel Hill, Chapel Hill, NC 27599 USA; 2 Department of Biostatistics, University of Washington, Seattle, WA 98195 USA; 3 Department of Epidemiology, University of North Carolina at Chapel Hill, Chapel Hill, NC 27599 USA; 4 Division of Public Health Sciences, Fred Hutchinson Cancer Center, Seattle, WA 98109 USA; 5 Department of Genetics, Rutgers University, New Brunswick, NJ 08901 USA; 6 Department of Statistics, Rutgers University, New Brunswick, NJ 08901 USA; 7 Vanderbilt Genetics Institute, Vanderbilt University Medical Center, Nashville, TN 37232 USA; 8 Department of Biostatistics, Vanderbilt University Medical Center, Nashville, TN 37232 USA; 9 Department of Medicine, Johns Hopkins University, Baltimore, MD 21287 USA; 10 Department of Epidemiology, University of Washington, Seattle, WA 98195 USA; 11 Division of Biostatistics, Institute for Health and Equity, and Cancer Center, Medical College of Wisconsin, Milwaukee, WI 53226 USA; 12 Division of Genetic Medicine, Department of Medicine, Vanderbilt University Medical Center, Nashville, TN 37232 USA; 13 Department of Genetics, University of North Carolina at Chapel Hill, Chapel Hill, NC 27599 USA

**Keywords:** Statistical methods, Genetic markers, Quantitative trait

## Abstract

Polygenic risk scores (PRS) have shown successes in clinics, but most PRS methods focus only on participants with distinct primary continental ancestry without accommodating recently-admixed individuals with mosaic continental ancestry backgrounds for different segments of their genomes. Here, we develop GAUDI, a novel penalized-regression-based method specifically designed for admixed individuals. GAUDI explicitly models ancestry-differential effects while borrowing information across segments with shared ancestry in admixed genomes. We demonstrate marked advantages of GAUDI over other methods through comprehensive simulation and real data analyses for traits with associated variants exhibiting ancestral-differential effects. Leveraging data from the Women’s Health Initiative study, we show that GAUDI improves PRS prediction of white blood cell count and C-reactive protein in African Americans by > 64% compared to alternative methods, and even outperforms PRS-CSx with large European GWAS for some scenarios. We believe GAUDI will be a valuable tool to mitigate disparities in PRS performance in admixed individuals.

## Abstract (web summary)

Most polygenic risk score (PRS) methods focus only on individuals with distinct primary continental ancestry, without accommodating recently-admixed individuals. Here, the authors develop a novel penalized regression-based PRS method specifically designed for admixed individuals.

## Introduction

Polygenic risk scores (PRS) have been successfully incorporated into clinical risk models for therapeutic interventions and disease screening^1–3^. However, PRS in personalized medicine disproportionately benefit European ancestry populations^4^ due to the severe under-representation of non-European ancestry individuals in genetic studies^5^. PRS calculated based on weights derived from European ancestry populations have consistently proven less predictive in non-European populations, and particularly poorly in individuals with substantial African ancestry^4,6^. Moreover, genetic admixture further complicates PRS transferability^6–8^ due to the unique and complex mosaic structure of chromosomal segments from different ancestral populations in admixed individuals, impeding the clinical utility of PRS in global populations.

Realizing the limitations of existing PRS in under-represented non-European ancestry populations, multiple statistical methods have been proposed to fill in the gap. Some attempt to borrow information from fine-mapping results and/or epigenetic annotations to construct PRS with variants that are more likely to be causal or functional across global populations^9–12^. These methods rely on the underlying assumption that causal variants are shared across populations with identical or similar effects. Under this assumption, PRS constructed with these causal variants are more transferable than standard PRS constructed with associated variants that are likely linkage disequilibrium (LD) tags of the causal variants. However, this assumption is rather strong and will miss variants with strong ancestral differential or specific effect, including variants with strongly ancestry-differentiated allele frequencies. For example, the Duffy null variant (rs2814778) residing in Atypical Chemokine Receptor 1 (*ACKR1*) gene, explains 7% variation in white blood cell count (WBC) among African Americans (AA)^13,14^. rs2814778 has high frequency (82.2%) among AAs and in many regions of West Africa^15^, while being essentially monomorphic (minor allele frequency <0.6%) in gnomAD^15^ and the 1000 Genomes Project^16^ in European populations. Other researchers explored cross-population PRS methods leveraging information from large-scale multi-ancestry or European ancestry GWAS studies to construct more ancestry-transferable PRS, either directly combining PRSs constructed separately from ancestry-specific GWAS^10,12,17,18^, or re-estimating effect sizes of GWAS variants when constructing PRS for non-European ancestry individuals^18–21^. These methods could theoretically allow some population-specific variants, but still suffer from power loss especially when the non-European sample sizes in GWAS are small, leading to biased effect estimates towards large-scale European ancestry individuals.

Importantly, although multiple methods have been developed for these under-represented populations^9,10,17–21^, few exist to explicitly model information from recently admixed populations. This is a missed opportunity because these admixed individuals provide valuable information regarding ancestry-specific effects, in mosaic chromosome segments. Approaches building on the mosaic structure of the genomes for recently admixed populations have been proposed. These methods aim to build better PRS by first disentangling such mosaics through local ancestry inference and then taking inferred local ancestry into PRS construction. For example, Marnetto et al. proposed the idea of partial PRS (pPRS)^7^. They first partitioned the whole genome into segments based on local ancestry, and then applied effect sizes from ancestry-specific GWAS in a piecewise manner across the genome to construct ancestry-specific PRS based on local ancestry partition, and finally combined these ancestry-specific PRSs. Bitarello and Mathieson provided some variations of this method by manipulating different weighing strategies in the final step to combine ancestry-specific PRSs^8^. These approaches are similar to each other and all have the following limitations. First, although causal variants are largely shared and have concordant effect sizes across populations^22^, these methods tend to ignore the complicated LD across EUR and AFR segments. In addition, GWAS studies tend to be biased toward discovering variants that are common in the population of the studied cohorts^4^. Therefore, though incorporating local ancestry estimates into PRS construction, these methods are still biased toward SNPs that are common in the ancestral populations well represented in the original GWAS studies.

In this work, we present GAUDI (Genetic Ancestry Utilization in polygenic risk scores for aDmixed Individuals), a novel penalized regression based PRS method developed specifically for admixed individuals that explicitly models ancestry-differential effects while borrowing information across ancestral segments in admixed genomes. Unlike previous methods, GAUDI does not necessarily rely on the use of external large-scale GWAS results, and can enhance prediction accuracy with moderate training sample size. Moreover, it can model PRS with high accuracy in the presence of ancestry-differential effects by balancing fusion and sparsity penalties in a fused lasso^23^ framework (Methods). By extensive simulation studies and comprehensive real data analysis, we demonstrate the benefits of GAUDI in admixed populations. We also show that GAUDI with moderate training sample sizes could sometimes outperform methods utilizing large-scale European GWAS results. We hope that our method can motivate researchers to develop more PRS methods tailored specifically for recently admixed populations to benefit the community and mitigate potential further exacerbations of existing health disparities from the use of poorly performing PRS in admixed populations.

## Results

### GAUDI overview

**Fig. 1:**
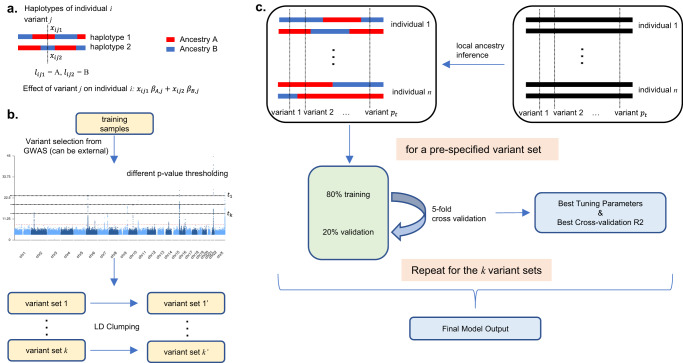
Overview of GAUDI model and framework. **a** Model set-up of GAUDI. Consider the haplotypes of individual *i* at variant *j* and assume local ancestry is already inferred. We consider the scenario with only two ancestries, namely A and B. Let ${x}_{{ij}1},{x}_{{ij}2}$ denote haplotype value (taking values 0 or 1 for a directly genotyped variant, and ranging from 0 to 1 for an imputed variant). Let ${l}_{{ij}1},{l}_{{ij}2}$ denote the local ancestry; here we have ${l}_{{ij}1}=A,{{l}}_{{ij}2}=B$. Let ${\beta }_{A,j},{\beta }_{B,j}$ denote population A, B specific effect of variant *j* on the phenotype. Thus we have the total effect of variant *j* in individual *i* as ${x}_{{ij}1}{\beta }_{A,j}+{x}_{{ij}2}{\beta }_{B,j}$. **b** Variant selection framework of GAUDI. We first perform GWAS or use external GWAS results to obtain *p*-values, which will be used for variant selection. Specifically, we use the thresholding strategy to identify variants that are marginally associated with the trait of interest at *k* pre-specified *p*-value thresholds, $\left({t}_{1},\cdots,{t}_{k}\right)$. These *k* sets of variants will be generated, and we then perform LD clumping for each of the *k* sets to both reduce dimension and remove variants in high LD. **c** Final PRS construction of GAUDI. After inferring the local ancestry for every participant in the training set, for a specific set of ${p}_{t}$ variants, we perform five-fold cross-validation to select the best tuning parameters, under the penalized regression framework. Repeating the process for the *k* variant sets and comparing the cross-validated R^2^ will give us the final PRS model.

Consider a sample of *i* = 1*, …, n* individuals admixed from two ancestral populations, *A* and *B*. Note that here we assume two ancestries for presentation brevity, but our method in principle can be generalized to multiple ancestries. Let ${y}_{1},\,\ldots,\;{y}_{n}$ denote phenotypic values for the individuals, ${x}_{{ij}1},{x}_{{ij}2}$ denote the phased allelic value (taking values 0 or 1 for typed markers and continuous values from 0 to 1 for imputed markers), and ${l}_{{ij}1},{l}_{{ij}2}$ denote the local ancestry, of individual *i* for SNP *j* on each of the two haplotypes (Fig. 1a). We model the phenotype as

1 $${y}_{i}=	\mathop{\sum }\limits_{j=1}^{p}[{\beta }_{A,j}({x}_{ij1}I({l}_{ij1}=A)+{x}_{ij2}\,I({l}_{ij2}=A)) \\ 	+{\beta }_{B,j}({x}_{ij1}I({l}_{ij1}=B)+{x}_{ij2}I({l}_{ij2}=B))+{\varepsilon }_{i}]$$

where *p* is the total number of SNPs, and $I\left(\bullet \right)$ is the indicator function. Under this model, ${\beta }_{A,j},{\beta }_{B,j}$ are the population *A, B* specific effect of SNP *j* on the phenotype. We note that with no local ancestry information, and ignoring haplotype information, this model collapses to the standard additive model.

Let ${{{{{{\bf{Y}}}}}}}_{n\times 1}$ denote the phenotype vector for *n* individuals, ${{{{{{\bf{G}}}}}}}_{n\times 2p}$ denote the haplo-genotype matrix, and ${{{{{{\boldsymbol{\beta }}}}}}}_{2p\times 1}$ denote the vector of effect sizes (Methods), we obtain estimated effect sizes via the following equation:

2 $$\hat{{{{{{\boldsymbol{\beta }}}}}}}(p,{{{{{\rm{\lambda }}}}}},{{{{{\rm{\gamma }}}}}})={{{{{{\rm{argmin}}}}}}}_{{{{{\beta }}}}} \frac{1}{2}\parallel {{{{{\bf{Y}}}}}}-{{{{{\bf{G}}}}}}{{{{{\boldsymbol{\beta }}}}}}{\parallel }_{2}^{2}+{{{{{\rm{\lambda }}}}}}\parallel {{{{{{\bf{D}}}}}}}_{3p\times 2p}{{{{{\boldsymbol{\beta }}}}}}{\parallel }_{1}$$

Terms inside argmin specify the fused lasso^23^ objective function where the penalty matrix **D** includes both fusion and sparsity components (Methods). The fusion component encourages similar ancestry-specific effects for the same variant, and the sparsity component penalizes inclusion of too many variants.

We also adopt the thresholding strategy for variant selection commonly used by PRS construction methods and perform LD clumping for each SNP set to both remove highly correlated SNPs and reduce computational burden (Methods, Fig. 1b). To ensure stable inference, we adopt cross-validations to choose tuning parameters, including ${{{{{\rm{\lambda }}}}}},{{{{{\rm{\gamma }}}}}}$ and the number of SNPs *p* (Methods, Fig. 1c). We then calculate PRS for each target individual with the estimated parameters.

### Simulation results

We evaluated the performance of GAUDI through comprehensive simulations by comparing its performance with the clumping and thresholding method implemented in PRSice^24^ and the previously proposed partial PRS (pPRS)^7^ method. We first performed small-scale proof-of-concept simulations using COSI^24^, with 102,572 genetic variants for 3500 AA individuals, and independent samples of 2500 EUR and 2500 AFR individuals serving as reference. We considered three different genetic settings of the causal variants in terms of their minor allele frequency (MAF) across ancestries: variants with EUR-MAF and AFR-MAF both >=5% (causal variants common in both ancestries), variants with EUR-MAF >=5% and AFR-MAF <5% (casual variants common only in EUR), and variants with EUR-MAF <5% and AFR-MAF >=5% (causal variants common only in AFR). For each of three MAF settings, we varied the proportion of causal variants to be 1, 0.5, and 0.05 to represent different polygenicity situations, the proportion of causal variants that have ancestry-identical effects to be 1 (no ancestry-differential effects) or 0.5, and the proportion of variation explained by genetic variations (i.e., heritability, h^2^) to be 0.2 or 0.6. In addition, we also varied the maximum LD R^2^ among causal variants to be 0.2 or 0.5. For PRS methods, we ran PRSice, pPRS, GAUDI with and without LD clumping for comparison (Methods).

Under all the different scenarios, GAUDI outperformed PRSice and pPRS across all simulated traits in the held-out testing data (Supplementary Note, Supplementary Figs. 1–3, Supplementary Data 1). Comparing across different polygenicity and heritability scenarios, GAUDI achieved best performance across the entire spectrum assessed, demonstrating most pronounced performance gains in settings with higher heritability and denser genetic architecture. The advantage of GAUDI is more pronounced in some scenarios with the introduction of ancestry-specific effects (Supplementary Note). In addition, the R^2^ attained by GAUDI in the testing dataset is nearly equal to heritability in almost all simulated phenotypes, demonstrating the power of GAUDI by borrowing information from haplotype segments in one ancestry to better estimate the effects in another ancestry.

**Fig. 2:**
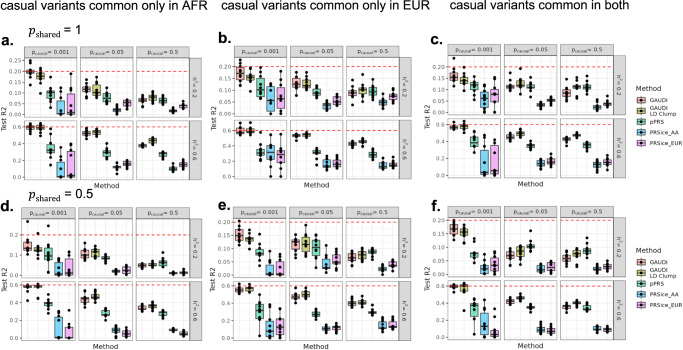
GAUDI performance compared to PRSice and pPRS in large EUR simulation studies under different settings. **a**–**c** ${p}_{{{\mbox{shared}}}}$ (proportion of variants with shared effects across ancestry groups) = 1: no ancestry-differential effects for all causal variants. **d**–**f** ${p}_{{{\mbox{shared}}}}$ = 0.5: half of the causal variants have ancestry-differential effects. **a**, **d** Causal variants are common only in AFR ancestry, specifically EUR-MAF < 5% and AFR-MAF > = 5%. **b**, **e** Causal variants are common only in EUR ancestry, i.e., EUR-MAF > = 5% and AFR-MAF < 5%. **c**, **f** Causal variants are common in both ancestries, i.e., EUR-MAF and AFR-MAF both >= 5%. Each experiment was repeated 10 times (shown in the box plots). The minima, maxima and center represent the minimum, maximum and median test R^2^ across the 10 repeats. The bounds of the boxes represent upper and lower quartiles, with whiskers represent 1.5 times of the interquartile range. The maximum LD R^2^ between causal variants were set to be 0.2 for all settings. The dashed red line denotes heritability. ${p}_{{{\mbox{causal}}}}$: proportion of causal variants out of all variants. Source data are provided as a Source Data file.

To mimic the realistic situation where large EUR cohorts are available compared to AFR and/or admixed AA individuals, we additionally included simulation settings with more individuals and more genetic variants. Specifically, we simulated 3,920 AA individuals with varying degrees of admixture (Supplementary Fig. 4) to accommodate the variation among admixed individuals, along with 48,000 EUR reference individuals. The total genetic variants simulated was ~4.2 million, and the simulation parameters were same as the above setting, except for the proportion of causal variants now changed to be 0.001, 0.05 and 0.5 (Methods). We similarly ran pPRS, PRSice with AA or EUR GWAS, and GAUDI with and without LD clumping. We found that GAUDI outperforms PRSice that uses GWAS results either from the same AA training individuals or from the much larger number of EUR individuals in every scenario, with an average improvement of 0.084 and 0.070 in R^2^ for scenarios with h^2^ = 0.2 and an average improvement by 0.35 and 0.34 for scenarios with h^2^ = 0.6, for PRSice-AA and PRSice-EUR, respectively. We note that under the situation of causal variants common only in AFR, PRSice-EUR sometimes performs even worse than PRSice-AA, despite utilizing the much larger sample size. The overall improvement of GAUDI is less pronounced when comparing with pPRS that leverages information from both AA and EUR reference samples, with an average improvement of 0.021 in R^2^ when h^2^ = 0.2, and an average improvement of 0.16 when h^2^ = 0.6 (Fig. 2). With the help of large EUR GWAS in some low heritability and high polygenicity scenarios, pPRS can perform better than GAUDI (Fig. 2f). For example, when the proportion of shared causal variants is 50%, proportion of causal variants is 5%, causal variants common in both populations and h^2^ = 0.2, the average R^2^ of pPRS is 0.04 higher than GAUDI, likely due to efficiently borrowing information from large EUR GWAS. However, the performance of pPRS under low polygenicity scenarios (0.001) is not satisfying, with the average R^2^ 0.066 and 0.22 lower compared to GAUDI in scenarios where h^2^ = 0.2 and 0.6, respectively. These results show that GAUDI may still help improve the PRS performance even when large EUR GWAS is available.

### Internal evaluations of GAUDI in WHI African Americans

We then performed real data analysis for African American (AA) from the Women’s Health Initiative (WHI) study. Here AA was defined with local ancestry information (Methods). We included 6,734 AA individuals from WHI PAGE GWAS project, and the majority of these AA individuals have a global African component greater than 50% of the genome (Supplementary Fig. 5). We additionally included 5,681 EUR individuals from WHI WHIMS + GWAS project as an ancillary cohort where EUR summary statistics are needed (Methods). We added PRS-CSx in our method comparison given its popularity in recent PRS literature^9,10,17–21^, along with PRSice and pPRS. We considered nine continuous phenotypes including white blood cell count (WBC), platelet count (PLT), hematocrit (HCT), hemoglobin (HGB), C-reactive protein (CRP), diastolic blood pressure (dBP), systolic blood pressure (sBP), body mass index (BMI) and serum creatinine, as well as three binary diseases including hypertension, stroke and type II diabetes (T2D). We partitioned the 6734 AA individuals into five folds and repeated the analysis five times with each fold as target set and the remaining samples as training set. Note that this outer-loop five-fold cross-validation is only for evaluation purpose and is not part of the GAUDI training step. For each of the repeats, GAUDI still utilizes five-fold cross-validation for the training samples (4/5 of all the samples) to avoid over-fitting.

**Fig. 3:**
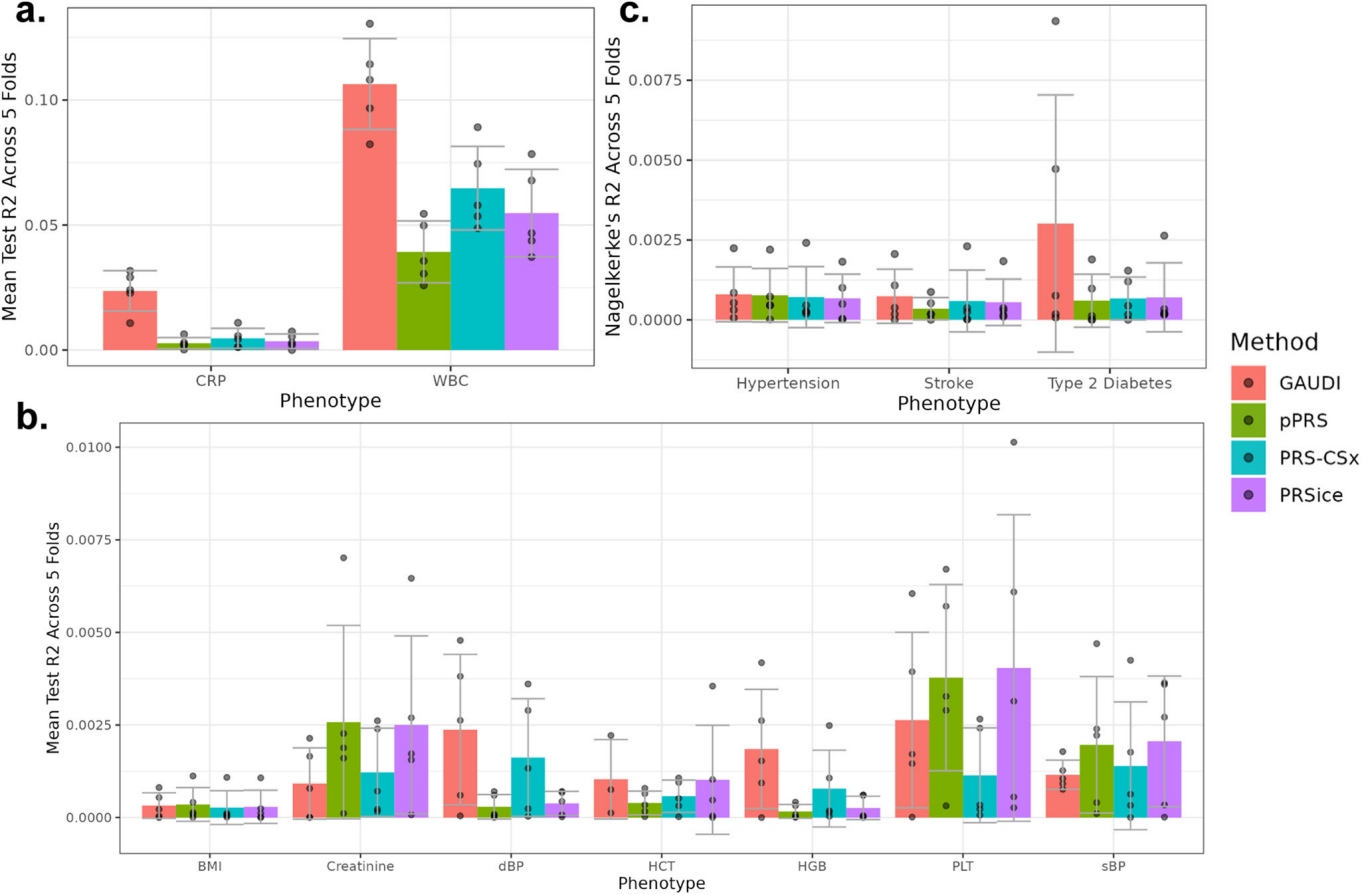
GAUDI performance compared to pPRS, PRS-CSx and PRSice in WHI AA internal evaluations for different traits. **a** CRP and WBC; **b** other continuous traits; **c** binary diseases. Each analysis was repeated five times, using five different WHI AA training and testing sets. The center of each bar plot represents the mean R^2^ across five folds, with the error bar denoting the standard deviation across the five replicates. CRP C-reactive protein, WBC White blood cell count, BMI Body mass index, dBP Diastolic blood pressure, HCT Hematocrit, HGB Hemoglobin, PLT Platelet count, sBP Systolic blood pressure. Source data are provided as a Source Data file.

Across the nine continuous phenotypes, only CRP and WBC showed significant non-zero mean R^2^ (Fig. 3a). For CRP and WBC, GAUDI substantially improves prediction accuracy compared to alternative methods. For example, GAUDI could achieve testing R^2^ of 1–3% for CRP, while the other three methods result in almost negligible R^2^ ( < 1%). For WBC, where all the methods provide meaningful non-zero R^2^, GAUDI improves the relative PRS prediction of 63.8% compared to PRS-CSx, 93.4% compared to PRSice, and 169.7% compared to pPRS. Such improvements are striking especially given the fact that GAUDI only utilizes variants with GWAS p-value < 5e-5, while PRS-CSx and PRSice considered all variants evaluated in GWAS. For example, GAUDI modeled an average of only 65 variants across the five folds to construct WBC PRS, while PRS-CSx and PRSice used > 500,000 variants. These results demonstrate the advantage of GAUDI by allowing differential effects across ancestry, suggesting that explicit modeling of the genetic mosaicism in recently admixed populations can be much more rewarding and influential than simply including more variants in PRS construction.

To ensure that the unsatisfactory performance of PRS-CSx was not due to lack of fine-tuning procedures, we tuned the global shrinkage parameter (phi) with a small grid-search as recommended by the authors. Though the performance of PRS-CSx under different phi’s are slightly different, they remain significantly inferior to GAUDI (Supplementary Fig. 6). We then explored why GAUDI performs much better for CRP and WBC, especially given the much smaller number of variants used. Therefore, we compared the EUR- and AFR- specific coefficients for each variant in the final PRS formula of the two traits. The results show that most variants have identical or similar effects across ancestries, but there are some outliers showing strong ancestry differential effects (Supplementary Figs. 7, 8). For example, we identified one variant, chr1:159680395:G:A (hg38, rs9651048), that has an AFR-coefficient 0.13 but an EUR-coefficient only 0.02 for CRP, demonstrating substantial differential effects across ancestries (Supplementary Fig. 7). We further note that this variant has extremely low MAF in EUR (MAF = 0.05% in TOP-LD^25^ EUR) but is common in AFR (MAF = 19.8% in TOP-LD^25^ AFR). This variant, as well as its only AFR LD tag (defined as LD R^2^ > 0.9 in TOP-LD^25^ AFR) rs10494326 (LD R^2^ = 0.92), do not exist in the HapMap3 variant list used as references in PRS-CSx, which may partially explain the unsatisfying performance of PRS-CSx. We note that local ancestry at this variant (rs9651048) is significantly associated with CRP values under the additive model (two-sided t-test p-value = 1.2e-6, Supplementary Fig. 9). Similarly, we identified some outliers for WBC (Supplementary Fig. 8, Supplementary Note), where GAUDI European weights are higher than GAUDI African weights. Further investigations show that the local ancestry at the two variants seems to be associated with the phenotype, where individuals with at least one copy of European local ancestry alleles tend to have higher values of white blood cell counts than those with both alleles of African local ancestry (Supplementary Fig. 10). In this sense, the weights from GAUDI shall be interpreted differently from how we typically interpret variant effect sizes, because the weights here change the predicted values of white blood cell counts on top of the linear combinations of other variant and local ancestry combinations. The advantages of GAUDI over other methods are likely due to its ability to capture such unusual phenomenon.

**Fig. 4:**
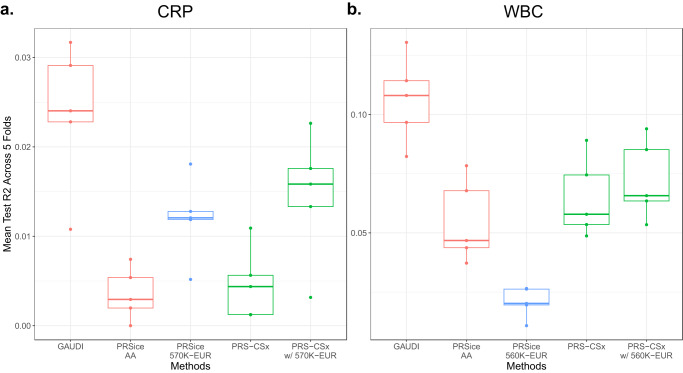
Performance comparison with PRS-CSx and PRSice utilizing large external EUR-based GWAS summary statistics. **a** CRP; **b** WBC. We compared GAUDI trained on ~5500 WHI AA individuals, with (1) PRSice using GWAS from the same ~5500 WHI AA training individuals; (2) PRSice using large external EUR-based GWAS (*N* > 560 K); (3) PRS-CSx using GWAS from both the same ~5500 WHI AA training individuals and WHI WHIMS + EUR-based GWAS (*N* = 5681); (4) PRS-CSx using GWAS from both the same ~5500 WHI AA training individuals and large external EUR-based GWAS (*N* > 560 K). Each analysis was repeated five times (shown in the box plots), using five different WHI AA training and testing sets. The minima, maxima and center represent the minimum, maximum and median test R^2^ across the 5 folds. The bounds of the boxes represent upper and lower quartiles, with whiskers represent 1.5 times of the interquartile range. The red color indicates methods that only used AA-based information; the blue color indicates methods that only used EUR-based information; and the green color indicates methods that used both AA- and EUR- based information. Source data are provided as a Source Data file.

Encouraged by the striking advantages of GAUDI, we next replaced the ancillary EUR GWAS from WHI WHIMS + GWAS (*N* = 5681) with large-scale meta-analysis results for CRP (*N* = 575,531)^26^ and WBC (*N* = 563,085)^27^ respectively, to investigate whether the superiority of GAUDI could be offset by the employment of large EUR GWAS results by alternative methods. Specifically, we compared GAUDI utilizing the same training samples (~5500 AA individuals) with PRSice and PRS-CSx, both of which harness the power of GWAS results from >500,000 EUR individuals. Strikingly, GAUDI still shows the best performance compared to these methods (Fig. 4). For CRP, PRSice and PRS-CSx perform significantly better with the help of large EUR GWAS compared to their performances with WHIMS + GWAS, but remain inferior to GAUDI. The relative mean testing R^2^ improvement of GAUDI is 63.4% and 97.5% compared to PRS-CSx and PRSice with large EUR GWAS (Fig. 4a). For WBC, PRS-CSx shows only slightly better improvement after switching to large EUR GWAS, and PRSice with large EUR GWAS performs significantly worse than other methods (Fig. 4b), indicating that EUR GWAS does not help much for PRS in admixed individuals. The results are expected given that the Duffy null variant (rs2814778) is essentially monomorphic in EUR but explains 7% variation in WBC among AA^13,14^. These findings demonstrate that PRS methods developed for individual level data, like GAUDI, are still valuable, complementary to summary-statistic-based methods that utilize large-scale EUR GWAS.

For other continuous and binary traits we tested, the prediction accuracies, measured by R^2^ for continuous traits and partial Nagelkerke’s R^2^ (Methods) for binary traits, are all very close to zero (Fig. 3b, c). The relative order and magnitude of prediction accuracy we observed are expected given the small training sample sizes and the difficulties of prediction in admixed individuals, consistent with recent applications of PRS to blood cell traits in AA samples with AFR weights^28^. Though the numerical results show that GAUDI achieves similar performance compared to other methods for traits without known large ancestry-differential effects, these comparisons should be interpreted with caveats due to the sample size limitations and prediction accuracies not significantly different from zero.

### External evaluations of GAUDI with UKB African ancestry individuals

In the previous section, we compared the performances of different PRS methods using cross-validations, where training and testing samples are both from the same WHI study and thus have similar genetic background. But this setting is likely over-optimistic for most real-life scenarios. To investigate the performance of GAUDI using external training dataset, we leveraged data from 9354 UK Biobank (UKB) individuals with African ancestry (UKB AFR) as defined previously^29,30^. We performed GWAS on these individuals, and applied the corresponding GWAS results as input for PRSice, PRS-CSx and pPRS. For GAUDI, we also utilized the same individuals for PRS training. We tested in the WHI AA individuals as in the previous section, but now using PRSs constructed using UKB samples (Methods). We considered phenotypes that we have access to in both datasets, including WBC, HCT, HGB, PLT, CRP, serum creatinine, hypertension, stroke and T2D.

**Fig. 5:**
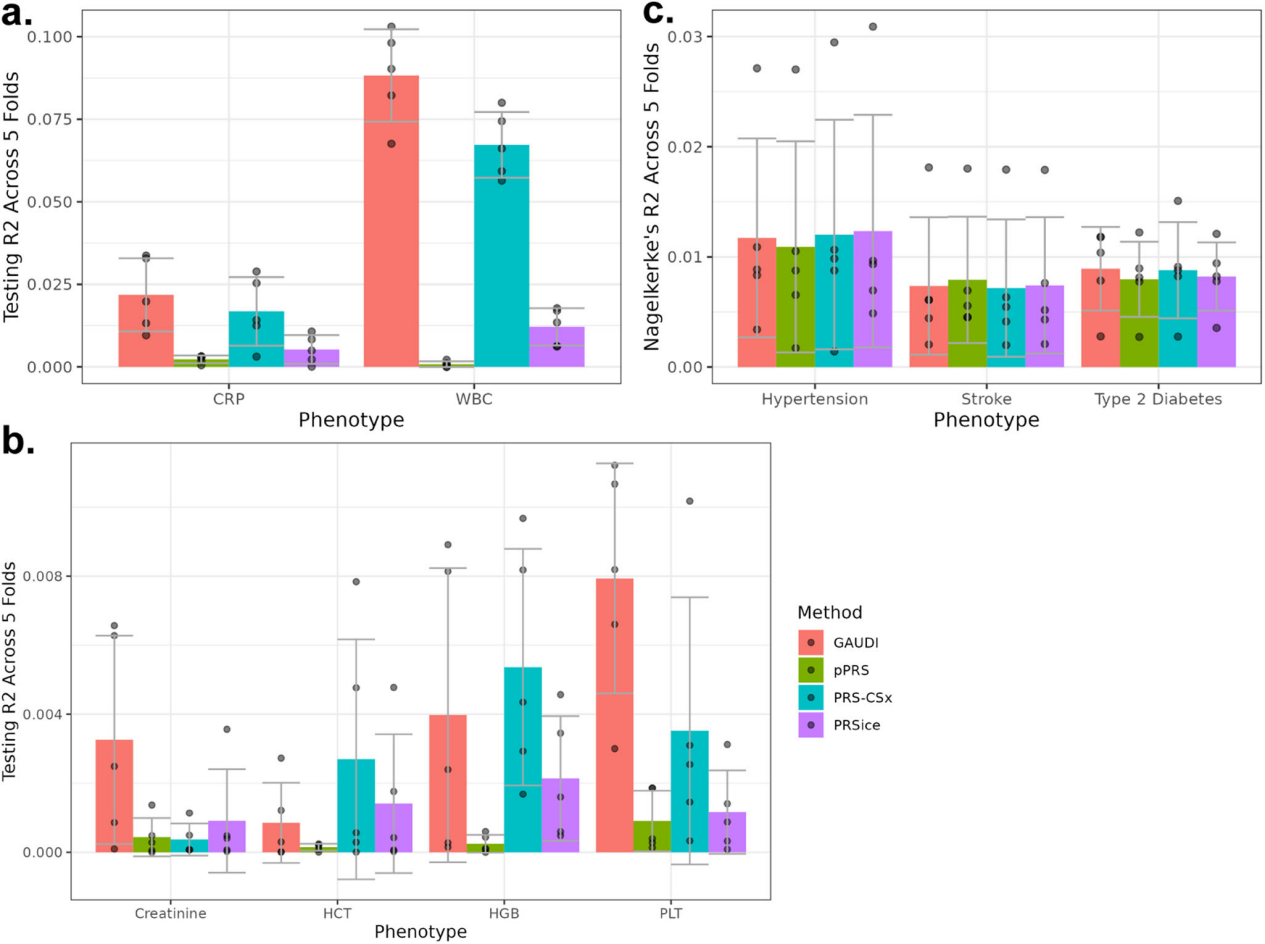
GAUDI performance compared to pPRS, PRS-CSx and PRSice in external evaluations for different traits. **a** CRP and WBC; **b** other continuous traits; **c** binary diseases. The models were trained using UKB participants with African ancestry, and applied to WHI AA individuals. Each analysis was repeated five times, using five different testing sets. The center of each bar plot represents the mean R^2^ across five folds, with the error bar denoting the standard deviation across the five replicates. CRP C-reactive protein, WBC White blood cell count, HCT Hematocrit, HGB Hemoglobin, PLT Platelet count. Source data are provided as a Source Data file.

We first note that there exists heterogeneity in the distributions of global African ancestry component between UKB AFR and WHI AA (Supplementary Fig. 11), where the UKB dataset contains over 3,000 individuals with ~100% AFR ancestry with no or little admixture. Even with the shifted genetic background, the advantages of GAUDI for CRP and WBC still hold (Fig. 5a). Compared to PRS-CSx, the relative improvement of GAUDI is 29.8% and 31.1% for CRP and WBC, respectively. The improved performance of PRS-CSx is likely because the UKB AFR training set matches better with the 1000 G^16^ AFR LD reference panel. For other continuous traits, all the R^2^’s are again essentially 0 (Fig. 5b); and all the methods perform highly similarly for the three binary diseases (Fig. 5c). We conclude that GAUDI still shows comparable or better performances even with external training samples.

### Trait screening in UKB: comparison with PRS-CSx under situations unfavorable for GAUDI

We showed previously that GAUDI demonstrated marked improvements only for CRP and WBC in our WHI AA targets. We would like to systematically identify other traits that benefit from GAUDI, compared to PRS-CSx utilizing much better-powered GWAS. Specifically, we leveraged data from UKB and screened 28 serum and urine traits, focusing on UKB AFR individuals as previously mentioned. We chose these quantitative traits for our evaluations as we anticipated better power to achieve non-zero predictive performance than for binary traits, which in most situations are limited in power to detect significant loci in UK Biobank alone. We similarly adopted outer-loop five-fold cross validations for method comparison, with each GWAS performed for different training samples. EUR GWAS summary statistics used by PRS-CSx were obtained from ~430,000 UKB EUR individuals. We then compared GAUDI using ~7000 AFR training samples with PRS-CSx using ~7000 AFR as well as ~430,000 EUR GWAS, an unfair situation for GAUDI in terms of GWAS discovery powers. As a benchmark, we also added PRSice using the same ~7000 AFR GWAS.

As expected, for 23 of the 28 traits screened, PRS-CSx achieved higher average R^2^ compared to GAUDI (Supplementary Data 2). However, for 4 of the remaining 5 traits where GAUDI shows higher R^2^, the difference in prediction accuracy is much higher (Table 1). For example, GAUDI achieved an average R^2^ of 9.2% for lipoprotein A, while PRS-CSx’s average R2 was merely 0.3%, despite the fact that it additionally leveraged the large UKB EUR GWAS. For another example, GAUDI achieved an average R^2^ of 2.1% and 3.8% for direct and total bilirubin, while the R^2^’s of PRS-CSx were only 0.01% and 0.04%, two orders of magnitude difference. Compared with PRSice, GAUDI shows higher average R^2^ for 19 out of 28 traits with the largest loss only 0.3% in R^2^ (Supplementary Data 2).

**Table 1:** Prediction accuracies for GAUDI, PRSice and PRS-CSx for the five traits that GAUDI shows higher average R^2^ compared to PRS-CSx in UKB quantitative serum and urine biomarker trait screening

Phenotype	GAUDI R^2^ Mean (sd)	PRSice R^2^ Mean (sd)	PRS-CSx R^2^ Mean (sd)
Lipoprotein A	0.092 (0.051)	4.6E-4 (3.5E-4)	3.0E-3 (2.8E-3)
Direct Bilirubin	0.021 (8.9E-3)	3.0E-3 (4.7E-3)	1.2E-4 (7.3E-5)
Total Bilirubin	0.038 (0.013)	4.8E-4 (5.0E-4)	4.5E-4 (4.2E-4)
Apolipoprotein B	0.050 (0.017)	0.016 (0.031)	0.026 (2.9E-3)
LDL direct	0.032 (4.8E-3)	4.8E-4 (2.3E-4)	0.030 (4.3E-3)

We note that GAUDI and PRSice constructed PRS using the same training samples (N ~ 7000), while PRS-CSx additionally leveraged large scale UKB EUR GWAS (N ~ 430,000). The R^2^ was calculated as the average of the five-fold outer-loop cross-validation R^2^. Comparison for a complete list of traits is provided in Supplementary Data 2.

We further assessed the estimated AFR- and EUR- specific weights from GAUDI for lipoprotein A, direct bilirubin and total bilirubin. We found that the total number of variants included in GAUDI PRS was < 100 for lipoprotein A, and some of the variants show ancestral-differential weights (Supplementary Fig. 12). In contrast, the number of variants included in GAUDI PRS for bilirubin traits was > 5000, suggesting that direct and total bilirubin may be more polygenic than lipoprotein A. Interestingly, there were obvious outliers in terms of effect size, rs1976391 for direct bilirubin and rs35754645 for total bilirubin. Specifically, these variants show much higher weights than other variants, and the AFR-weights more than doubles the EUR-weights (0.19 v.s. 0.08 for rs1976391 for direct bilirubin, 0.19 v.s. 0.05 for rs35754645 for total bilirubin) (Supplementary Figs. 13, 14). These examples, along with the aforementioned Duffy null variant example, though surprising, represent interesting and important examples of the genetic architectures of different traits when involving diverse ancestry populations and warrant more attention and methods tailored to admixed populations.

## Discussion

PRS have been given unprecedented yet warranted attention recently, but the performance in diverse non-EUR populations is still quite inferior to EUR, especially poorly in admixed African individuals. Multiple PRS methods have been proposed for improving PRS performance in non-EUR populations, but most still focus on individuals with one single primary genetic ancestry, without considering admixed individuals. In this study, we developed a novel PRS method, GAUDI, that targets specifically for admixed individuals, by jointly modeling ancestry-specific effects via a penalized regression framework to account for the unique mosaic structure of the genetic segments for these individuals. Note that we named our method after Antoni Gaudí, a Catalan architect from Spain known for a special mosaic design technique, with the rationale that our method is also designed for mosaic structure, specifically genetic mosaicism in admixed genomes.

Our model is a natural extension from the standard PRS model when considering only one shared effect across ancestry, but is more flexible to allow for ancestry-differential effects. GAUDI demonstrates substantial advantages over other methods for CRP and WBC, traits for which variants showing ancestry-differential effects exist, and the superiority remains even when compared to other methods that use large-scale EUR GWAS summary statistics. We evaluated more traits by screening serum and urine biomarkers in UKB, and found that GAUDI shows similar advantages for lipoprotein A, direct and total bilirubin by allowing ancestral-differential effects. While these are extreme examples, the ability of GAUDI to capture such extremes is clinically meaningful. A recently publication shows that the Duffy null variant (rs2814778) should be accounted for in clinical decision-making to avoid unnecessary bone marrow biopsy procedures^31^. In addition, we also showed that GAUDI performs similarly to alternative methods when applying to other complex traits and diseases (e.g., blood pressure, platelet count, etc.) for which no known variants exhibit large ancestry-differential effects, in the situation of using similar GWAS sample sizes for discoveries. We note that not all traits benefit from GAUDI, especially when comparing with methods like PRS-CSx that leverage much better-powered GWAS summary statistics to construct PRS. We are not developing a PRS method that performs the best for every single trait, arguably there is no such method now or ever. Our method provides a new and alternative framework for PRS by explicitly modeling local ancestry in PRS construction. We showed that GAUDI achieves enhanced performance for some traits, depending on the genetic architecture of traits. We argue that our method may not provide better performance for every single trait, but importantly it may help substantially in some scenarios, which could also allow us to gain insights into the genetic architecture for traits of interest and has the potential to uncover genetic variants that show ancestral differential effects. We believe GAUDI will be a valuable tool facilitating speedy translation of PRS in clinic.

GAUDI also provides a protocol to mitigate the mismatch between GWAS and PRS target cohorts. The genetic difference between large-scale GWAS and target populations is a plaguing issue for many of the PRS methods. Additionally, the differences between LD reference panels and target populations can further exacerbate the difference, which may be the reason why we observe unsatisfying performance of PRS-CSx for several traits. By contrast, in our GAUDI framework, we only use the p-value information from GWAS for variant screening purposes. The GWAS p-values for variant selection could be either from the same training cohort or from external GWAS that include individuals with the same or similar ancestral background as the target (e.g., the multi-ethnic GWAS) to ensure sufficient ancestry representation for the target. Caveats should be kept in mind that some multi-ethnic GWAS could remove variants that are rare in Europeans^32^, which was shown that some ancestry-specific variants are still missed^29^. Most other methods, in addition to GWAS p-values, use effect size estimates. In contrast, our effect sizes are directly estimated from the mosaic-structured admixed genomes to decipher potential ancestry-differential effects.

GAUDI utilizes local genetic ancestry information to directly model ancestry-differential effects, and the idea of leveraging local ancestry has previously been implemented in several methods including our LAAA^33^, and Tractor^34^, a recently published GWAS method. Though similarly integrating local ancestry, GAUDI is essentially different from LAAA and Tractor. Both of them model only one variant at a time, incorporate local ancestry in GWAS stage and provide marginal effect size estimates in single-variant tests separately for each ancestry. Therefore, we still need to solve the issues existing in PRS construction with GWAS summary statistics, for example, how to select variants, and how to account for LD, etc. In contrast, GAUDI incorporates local ancestry in the PRS modeling stage, where GWAS only serves for variant pre-screening purposes and the effect sizes are estimated jointly (jointly not only across ancestries, but also simultaneously for many variants). We note that the current implementation of GAUDI only considers two ancestries. Though theoretically generalizable to more ancestries, implementation and careful evaluation warrant separate future work.

GAUDI relies on individual level data for model training and adopts a fused lasso framework to jointly estimate ancestry-specific effects. It is therefore computationally more intensive than summary statistics based methods. The current implementation prohibits the inclusion of millions of variants. Practically, we recommend LD clumping before applying GAUDI. In our experiments, GAUDI could fit models using variants with p < 5e-5 ( ~ 1-2 K after LD clumping) within 1 h, but when relaxing the p-value threshold to 5e-4 (leading to ~15 K variants after LD clumping), the computational time increased to ~40 h (Supplementary Fig. 15). Improving computational efficiency and/or developing more efficient algorithms for parameter estimation are important future research directions.

We understand that individual-level-data-based PRS methods may be less flexible than summary-statistics-based methods, but our results show that efficiently leveraging individual level data can outperform summary-statistics-based methods that use results from EUR-centric GWAS of much larger sample sizes. While currently individual level data are rarely available to a given research team in sufficiently large sample sizes for optimal PRS construction, efforts which allow for broad data access and sharing (such as NIH’s All of Us) and newly formed consortia (such as NHGRI’s Polygenic RIsk MEthods in Diverse populations (PRIMED) consortium) strive to allow for such analyses at larger scale. We are investing efforts to implement that our GAUDI methodology in platforms that allow us to analyze these larger individual-level datasets for admixed individuals.

In summary, both comprehensive simulations and real data analysis demonstrate the superiority of GAUDI over alternative methods by allowing ancestry-differential effects, which we anticipate will be increasingly observed with larger numbers of non-European ancestry individuals evaluated in genetic association studies. Our strategy of allowing ancestry-differential effects provides a protocol to construct PRS in admixed individuals, and we point out some potential future directions to extend the model. We believe with more admixed individuals enrolled in more studies in the coming years, the community will benefit even more from GAUDI, particularly to avoid further exacerbating health disparity for admixed individuals.

## Methods

### Model setup

Consider the problem of constructing PRS for a sample of *i* = 1*, …, n* individuals recently admixed from two ancestral populations, *A* and *B*. This model can be extended to an arbitrary number of ancestral populations, but for simplicity here we consider only two ancestral populations. Let ${x}_{{ij}1},{x}_{{ij}2}$ denote the allelic value of individual *i* for variant *j* on haplotype 1 and 2, respectively (Fig. 1a), taking values 0 or 1 for genotype data, or ranging continuously from 0 to 1 for imputed dosages. Similarly, let ${l}_{{ij}1},{l}_{{ij}2}$ denote the local ancestry of individual *i* for variant *j* on haplotype 1 and 2, respectively, taking values A or B for the corresponding ancestral population. Let ${{{{{\bf{Y}}}}}}={\left({y}_{1},\cdots,{y}_{n}\right)}^{{\prime} }$ be an *n × *1 phenotype vector, and we assume

3 $${y}_{i} =	 \mathop{\sum }\limits_{j=1}^{p}[{\beta }_{A,j}({x}_{ij1}\,I({l}_{ij1}=A)+{x}_{ij2}I({l}_{ij2}=A)) \\ 	+ {\beta }_{B,j}({x}_{ij1}I({l}_{ij1}=B)+{x}_{ij2}\,I({l}_{ij2}=B))+\,{\varepsilon }_{i}]$$

where *p* is the total number of variants, and $I\left(\bullet \right)$ is the indicator function. A subset of the variants, ${p}^{*}$, are causal, meaning that the effect of the variants on the phenotype is non-zero. Under this model, ${\beta }_{A,j},{\beta }_{B,j}$ are the population *A, B* specific effect of variant *j* on the phenotype. With no local ancestry information, nor regards to haplotype information, this collapses to the usual genetic association model

4 $${y}_{i}=\mathop{\sum }\limits_{i=1}^{p}{x}_{ij}{\beta }_{j}+{{{{{{\rm{\varepsilon }}}}}}}_{i}$$

Where ${x}_{{ij}}$ is the allelic values of individual *i* for variant *j*, and ${\beta }_{j}$ is the effect size of variant *j*.

We further write the above model using matrix notation. Let ${x}_{{ijP}}={x}_{{ij}1}I\left({l}_{{ij}1}=P\right)+{x}_{{ij}2}I\left({l}_{{ij}2}=P\right)$ where *P* denotes population ancestry, taking values *A* or *B*. Then, the design matrix is given by

5 $${{{{{{\bf{G}}}}}}}_{n\times 2p}={\left(\begin{array}{ccccccc}{x}_{11A} & {x}_{11B} & {x}_{12A} & {x}_{12B} & \cdots & {x}_{1pA} & {x}_{1pB}\\ {x}_{21A} & {x}_{21B} & {x}_{22A} & {x}_{22B} & \cdots & {x}_{2pA} & {x}_{2pB}\\ \vdots & \vdots & \vdots & \vdots & \ddots & \vdots & \vdots \\ {x}_{n1A} & {x}_{n1B} & {x}_{n2A} & {x}_{n2B} & \cdots & {x}_{npA} & {x}_{npB}\end{array}\right)}_{n\times 2p}$$

We then define ${{{{{{\mathbf{\beta }}}}}}}_{2p\times 1}=\left({\beta }_{A,1},\,{\beta }_{B,1},\, \cdots,\;{\beta }_{A,p},\,{\beta }_{B,p}\right)$, thus the above phenotype model could be represented as ${{{{{\bf{Y}}}}}}={{{{{\bf{G}}}}}}\,{{{{{\mathbf{\beta }}}}}}+{{{{{\mathbf{\varepsilon }}}}}}$, where ${{{{{\mathbf{\varepsilon }}}}}}=\left({\varepsilon }_{1},\cdots,{{{{{{\rm{\varepsilon }}}}}}}_{n}\right)$ is the error vector.

The problem of PRS construction under this model is equivalent to the problem of accurate estimation of the population specific effects for ancestral populations *A* and *B* with the design matrix specified, and given that the predictors (variants) are already selected.

### GAUDI framework

Our GAUDI method for PRS construction for admixed individuals is a modified fused lasso approach. Specifically, given genotype information for *n* admixed individuals at *p* variants, some subset of which (denoted by ${p}^{*}$ as the number) are causal variants. We assume that for each individual we have also obtained haplotype-resolved local ancestry inference estimates via RFMix.

The variant selection workflow is shown in Fig. 1b. Using the training sample, we perform GWAS and select variants based on GWAS p-values. Note that it is also acceptable to use external GWAS results, which include individuals with similar ancestral background as the target (e.g., multi-ethnic GWAS), to select variants, which can be preferred for at least two reasons. First, we can leverage information from larger sample size and thus more powerful GWAS already carried out. Second, using external GWAS results will save computation costs for running GWAS in the training sample. With GWAS p-values, we adopt a grid search strategy to select variants. For *k* pre-specified *p*-value thresholds, $\left({t}_{1},\cdots,{t}_{k}\right)$, we can identify *k* sets of variants passing each of the thresholds. Then we perform LD clumping on each of the *k* selected variant sets to both reduce dimension and remove variants in high LD for more stable inference. Let ${p}_{t}$ denote the total number of variants for the set of variants selected with *p*-value threshold *t*. We then adopt a grid search strategy via five-fold cross validation to estimate the best tuning parameters using the following fused lasso objective function:

6 $$f({{{{{\mathbf{\beta }}}}}}|{{{{{\rm{\lambda }}}}}},{{{{{\rm{\gamma }}}}}},{p}_{t})=\frac{1}{2}\parallel {{{{{{\bf{Y}}}}}}}_{n\times 1}-{{{{{{\bf{G}}}}}}}_{n\times 2{p}_{t}}{{{{{{\mathbf{\beta }}}}}}}_{2{p}_{t}\times 1}{\parallel }_{2}^{2}+{{{{{\rm{\lambda }}}}}}\parallel {{{{{{\bf{D}}}}}}}_{3{p}_{t}\times 2{p}_{t}}{{{{{{\mathbf{\beta }}}}}}}_{2{p}_{t}\times 1}{\parallel }_{1}$$

where the penalty matrix **D** is given by

7 $${{{{{{\bf{D}}}}}}}_{3{p}_{t}\times 2{p}_{t}}=\left(\begin{array}{c}{{{{{{\bf{D}}}}}}}_{1}\\ {{{{{{\bf{D}}}}}}}_{2}\end{array}\right)=\left(\begin{array}{ccccccc}1 & -1 & 0 & 0 & \cdots & 0 & 0\\ 0 & 0 & 1 & -1 & \cdots & 0 & 0\\ \vdots & \vdots & \vdots & \vdots & \cdots & \vdots & \vdots \\ 0 & 0 & 0 & 0 & \cdots & 1 & -1\\ {{{{{\rm{\gamma }}}}}} & 0 & 0 & 0 & \cdots & 0 & 0\\ 0 & {{{{{\rm{\gamma }}}}}} & 0 & 0 & \cdots & 0 & 0\\ \vdots & \vdots & \vdots & \vdots & \cdots & \vdots & \vdots \\ 0 & 0 & 0 & 0 & \cdots & 0 & {{{{{\rm{\gamma }}}}}}\end{array}\right)$$

Then we compare the optimized performance for *k* variant sets with different p-value thresholds, and report the best one as the final constructed PRS model (Fig. 1c).

One notable difference between GAUDI and traditional fused lasso is that only ancestry-specific effects for a given variant are penalized with fusion, rather than all adjacent parameters. We finally calculate the PRS for a target sample using

8 $${{{{{{\rm{PRS}}}}}}}_{{{{{{\rm{target}}}}}}}={{{{{{\bf{G}}}}}}}_{{{{{{\rm{target}}}}}}}\hat{{{{{{\mathbf{\beta }}}}}}}$$

Cross-validated model performance for tuning parameters (${{{{{\rm{\lambda }}}}}},{{{{{\rm{\gamma }}}}}}$ and the *p*-value threshold ${t}_{i}$) is optimized based on the squared Pearson correlation between the observed phenotype and the PRS calculated above.

### COSI genotype simulations

In order to simulate haplotypes of recent admixture, we used COSI^24^ to generate six 500 kb regions for 3,500 AA individuals, with each region containing ~17k variants. The total number of variants simulated is 102,572. We made two primary assumptions in generating our simulated haplotypes. First, we assumed that the global ancestry proportions of our AA samples were 80% African and 20% European. Second, using empirical estimates of ancestral switch-points based on an analysis of TOPMed individuals^35^, we assumed 4% of 500Kb regions would contain ancestry switch-point events. Thus, for 3500 diploid individuals, 280 chromosomes contained switch points (7000 * 0.04 = 280). For each ancestry switch point chromosome, we generated one EUR and one AFR chromosome to simulate the admixture event at a random base-pair in the region. For the remaining 6,720 chromosomes with no admixture events, we generated 80% AFR chromosomes (*n* = 5376) and 20% European chromosomes (*n* = 1344). Additionally, we simulated 5,000 EUR chromosomes and 5,000 AFR chromosomes to be used as reference for relevant methods.

### Phenotype simulations

We simulated phenotypes using 500 kb regions generated from COSI simulated genotypes for the 3500 admixed individuals and the 2500 reference AFR and EUR individuals. We considered three distinct sets of causal variants to mimic different genetic architectures.

First, we created the “causal variants common in both ancestries” scenario. At a locus, we considered variants that had both AFR MAF and EUR MAF >=0.05 as candidate causal variants. Second, we created the “causal variants common only in EUR” scenario, where variants that had AFR MAF <0.05 and EUR MAF >=0.05 were considered as candidate causal variants. Third, we similarly created the “causal variants common only in AFR” scenario, where variants that had AFR MAF >=0.05 and EUR MAF <0.05 were considered as candidate causal variants. We note that the AFR and EUR MAF here refer to the ancestry-component-specific MAF from the admixed genomes, and we removed variants with both AFR and EUR MAF <0.5% when considering causal variants.

For a variant *j*, we simulated its effect sizes from the following distribution

9 $$\left\{\begin{array}{c}{\beta }_{A,j}={\beta }_{B,j}\sim N(0,1)\\ {\beta }_{A,j}\sim N(0,1),{\beta }_{B,j}\sim N(0,1)\\ 0\end{array}\begin{array}{cc}{{{{{\rm{with}}}}}}\,{{{{{\rm{probability}}}}}} & {p}_{{{{{{\rm{causal}}}}}}}{p}_{{{{{{\rm{shared}}}}}}}\\ {{{{{\rm{with}}}}}}\,{{{{{\rm{probability}}}}}} & {p}_{{{{{{\rm{causal}}}}}}}(1-{p}_{{{{{{\rm{shared}}}}}}})\\ {{{{{\rm{with}}}}}}\,{{{{{\rm{probability}}}}}} & 1-{p}_{{{{{{\rm{causal}}}}}}}\end{array}\right.$$

We changed the values of four different parameters to evaluate a wide spectrum of genetic architectures. First, we varied the proportion of causal variants (${p}_{{{\mbox{causal}}}}$), taking three possible values 0.05, 0.5 and 1, to represent different levels of polygenicity. Second, we varied the proportion of variants that have the same effect size across ancestry groups (${p}_{{{\mbox{shared}}}}$), taking three possible values 1, 0.8, 0.5, to represent varying extents of genetic heterogeneity across ancestries. Third, we varied heritability (${h}^{2}$), or the proportion of variation explained by genetic effects, taking possible values 0.2 or 0.6. Finally, we allowed different levels of maximum correlation between causal variants (${r}^{2}$), up to 0.2 and 0.5, to test GAUDI model stability in the presence of correlated causal variants.

For varying the LD between causal variants in the phenotype, we performed LD pruning on the set of candidate causal variants using PLINK (–indep-pairwise 500 5 ${r}^{2}$)^36^. We repeated each combination of the above parameters 10 times for each of the three causal variant scenarios. We simulated the error terms from the standard normal distribution.

### Simulations with large EUR references

We expanded our simulation settings to accommodate the scenario where large EUR GWAS references are available and where different admixed individuals have varying degrees of admixture. We first generated the proportion of AFR components from N(0.5, 0.003) distribution ignoring negative values, and then assigned AFR and EUR chromosomes according to the degrees of admixture and similarly assuming 4% switch-over events. We in total generated 3,920 AA individuals. We additionally simulated 2,000 AFR and 48,000 EUR individuals as references. In order to include more variants in a computationally efficient manner, we simulated 45 regions with each of 100 kb length and containing ~90 k variants. In total, we have 4,196,402 variants summed across the 45 simulated regions. We also changed the proportion of causal variants to be 0.001, 0.05 and 0.5 to allow the investigation of less polygenicity scenarios. Other phenotype simulation parameters were similar as previously mentioned. Specifically, we similarly created three different causal variant situations, namely causal variants common only in EUR, or only in AFR, or in both. We fixed *h*^2^ to be 0.2 or 0.6 and ${p}_{{{\mbox{shared}}}}$ to be 1, 0.8 or 0.5. We also set the maximum correlation between causal variants to be 0.2.

### The WHI cohort

The Women’s Health Initiative (WHI) is one of the largest (*n* = 161,808) studies of women’s health ever undertaken in the U.S. There are two major components of WHI: (1) a clinical trial (CT) that enrolled and randomized 68,132 women ages 50-79 into at least one of three placebo control clinical trials (hormone therapy, dietary modification, and supplementation with calcium and vitamin D); and (2) an observational study (OS) that enrolled 93,676 women of the same age range into a parallel prospective cohort study^37^. A diverse population including 26,045 (17%) women from minority groups was recruited from 1993 to 1998 at 40 clinical centers across the U.S. Details on the study design, eligibility, recruitment, and the reliability of the baseline measures of demographic and health characteristics have been published elsewhere^37,38^. Fasting blood samples were obtained from all participants at baseline and were analyzed for white blood cell count and platelet count by certified laboratories at each of the 40 clinical centers as part of a complete blood count (CBC)^38^. Results were entered into the WHI database at each clinical center and were reviewed by clinical center staff^39^. These assays were performed in a single laboratory using the same methods. CBCs were measured within 30 hours of blood draw. A detailed description of all the phenotype definitions has been published previously^40^.

The WHI PAGE GWAS project performed genotyping among self-identified non-Hispanic Black or African American (*n* = 6897) and Hispanic/Latino (*n* = 4754) women from WHI who consented to genetic research. These participants were genotyped by the Population Architecture using Genomics and Epidemiology (PAGE) study, along with participants of non-European ancestry from the Hispanic Community Health Study/Study of Latinos (HCHS/SOL), the Multiethnic Cohort (MEC), and the Icahn School of Medicine at Mount Sinai BioMe biobank (BioMe) (total MEGA sample size *n* = 49,839). Genotyping was performed using the Multi-Ethnic Genotyping Array (MEGA); quality control has been performed at both sample and variant level^40^ and included exclusion of variants based on high missingness, Mendelian error rates, discordant calls among study duplicate samples, and other filters. This array was designed to provide improved multi-ethnic coverage of common and low frequency variants, and also included custom content for fine-mapping GWAS loci and genotyping clinically reported and exonic variants^41^.

The WHI WHIMS + GWAS project performed genotyping among women of European descent with appropriate consent for genetic data sharing on dbGaP using the Illumina Omni Express platform. When these participants are combined with the GARNET (Genomics and Randomized Trials Network from NHGRI) participants (who were genotyped on the Illumina Omni-Quad chip), they form a population that is representative of the entire European American hormone trial population from WHI.

### Internal evaluations in WHI samples

In this study, we included 6734 AA individuals from the WHI PAGE GWAS study and 5,681 EUR individuals from WHI WHIMS + GWAS study after excluding individuals with missing phenotype or covariates, to compare the performance of GAUDI with PRSice, pPRS, and PRS-CSx. The EUR individuals were included as ancillary samples in order to apply pPRS and PRS-CSx, both of which require EUR GWAS estimates as input. We used 5-fold cross validation to assess performance of different methods.

Genotype imputation. Genotype imputation was performed with TOPMed freeze 8 reference panel^42^ following the procedure of our previous work^29,43–45^, using Eagle v2.4^46^ for phasing and minimac4^47^ for imputation. We performed imputation separately for AA samples or EUR samples. Starting from the genotype array data, we removed samples and variants with missingness > 10%, and then uploaded the data to TOPMed imputation server to perform imputation. After imputation, we re-calculated the estimated imputation quality (Rsq) to account for sample overlap with the reference panel, and performed post-imputation QC by including well-imputed variants with imputation Rsq > 0.3 for common variants (MAF > 1%) and imputation Rsq > 0.6 for low frequency variants (MAF in [0.1%, 1%]).

Phenotype processing. We considered nine continuous phenotypes (CRP, WBC, PLT HCT, HGB, BMI, dBP, sBP and serum creatinine) and three binary diseases (hypertension, stroke and T2D) with low levels of missing data. All phenotypes were adjusted by cohort for age, squared age, top 10 genotype PCs, recruitment center and genotyping array using linear regression models for continuous phenotypes and logistic regression models for binary traits. WBC values were log_10_(x + 1) transformed before regression. Residuals from the regression models were inverse normal transformed and served as the phenotypes for GWAS analysis.

GWAS. For the GWAS association tests, we considered common variants (MAF > 0.01) with Rsq > 0.3, and low frequency variants (MAF in (0.001, 0.01)) with Rsq > 0.6. Note that for our training samples, MAF = 0.001 corresponds to a minor allele count (MAC) of approximately 10. We performed GWAS using REGENIE^48^ separately for each of the five training sets of AA individuals (i.e., for 5-fold cross validation), and for all the EUR individuals, on the residuals of each phenotype. To fit the REGENIE null model accounting for cryptic relatedness, we used extremely-well imputed common variants (MAF > 0.2, Rsq > 0.9999). We fit these phenotypes simultaneously using the grouping options available in REGENIE with default parameters.

### External evaluations using UKB

We included 9,354 UKB participants with AFR ancestry, as defined in our previous work^29,30^. In brief, these individuals were defined with a strategy combining both self-reported race/ethnicity and *k*-means defined PC clusters. When comparing different PRS methods, we trained models using the UKB AFR individuals and tested on the WHI AA individuals. For methods where ancillary EUR GWAS are needed, we similarly provided the GWAS from WHI WHIMS + .

Genotype imputation. Similar to the WHI cohort, genotype imputation was performed with TOPMed freeze 8 reference panel^42^ using Eagle v2.4^46^ for phasing and minimac4^47^ for imputation. After imputation, we included variants with imputation Rsq >0.3 for common and low frequency variants (MAF >=0.5%) and variants with imputation Rsq > 0.8 for rare variants (MAF <0.5%).

GWAS. We previously have performed GWAS for these UKB AFR individuals for WBC^49^, HCT, HGB^50^, PLT^51^, CRP and serum creatinine^29^ using EPACTS^52^. To be consistent, we similarly performed GWAS for hypertension, stroke and T2D using the same software. Hypertension was defined with ICD 10 code I10 or ICD 9 code 401.0, 401.1 or 401.9; stroke was defined with ICD 10 code I63 or ICD 9 code 433, 434 and 436; and T2D was defined with ICD 10 code E11 or ICD 9 code 250.

### Local ancestry inference

For the AA samples in both simulations and real data analysis, we inferred local ancestry using RFMix^53^ with data from the 1000 Genomes Project (1000 G)^16^ as the reference panel. We considered only EUR and AFR ancestry since our analyses focused on AAs. Specifically, our 1000 G reference panel included 92 EUR samples and 92 AFR samples. For local ancestry inference, we kept only common variants with MAF >0.05.

### PRS method application

GAUDI. When applying GAUDI, we included variants that had a MAC >10 on at least one ancestral haplotype. If the variant was polymorphic in only one ancestral population, we included only one ancestry-specific effect in the model. If the variant was polymorphic in both populations, we included both ancestry-specific effects in the model. To reduce the number of variants in models, we first performed LD clumping with stringent *r*^2^ 0.1 threshold using in-sample LD to remove correlated variants, and then included all the clumped variants with *p* < 0.05 as input variant set for GAUDI. Other details of GAUDI were described previously in the GAUDI framework section. In simulation studies, we trained GAUDI models using only the simulated admixed individuals without borrowing information from any of the simulated reference samples. For real data analysis, similarly GAUDI models were trained on admixed training individuals (e.g., WHI AA or UKB AFR).

PRSice. PRSice is a popular software implementation of the P + T or C + T method^54^, a simple single-population PRS method. In small-scale simulation studies, we applied PRSice to the GWAS summary statistics from the reference AFR individuals, and then applied the formula (PRSice selected variants and their weights estimated from training samples) on testing samples to obtain the weighted sum, which was the PRS for testing samples. In simulations that include large EUR sample sizes, we applied PRSice in two ways: (1) to GWAS summary statistics from the training AA individuals (same individuals as used in GAUDI), which we refer to as PRSice-AA; and (2) to GWAS summary statistics from the large number of EUR individuals, referred to as PRSice-EUR. In real data analysis, we applied PRSice to the GWAS summary statistics from the training admixed individuals (e.g., WHI AA or UKB AFR) without borrowing information from European ancestry individuals. For each implementation, in-sample LD was calculated for training the PRS models. Note that p-value fine-tunning was performed every time when applying the PRSice method.

Partial PRS (pPRS). pPRS is a method to incorporate local ancestry information in PRS estimation in admixed individuals using only summary statistics from the ancestral populations^7^. Similar as in PRSice evaluation, in-sample LD was calculated in the training samples. In simulations, we applied pPRS with GWAS results from EUR and AFR reference samples. In the internal real data analysis with WHI, GWAS summary statistics were derived from WHI WHIMS + EUR and WHI AA training individuals; in the UKB real data analysis, GWAS results were obtained from UKB EUR and UKB AFR training samples; in the cross-cohort evaluation analysis between WHI and UKB, GWAS results were obtained from WHI WHIMS + EUR and UKB AFR.

PRS-CSx. PRS-CSx is a recently developed method that integrates GWAS summary statistics from multiple populations while accounting for LD from external reference panels to improve cross-population PRS prediction^18^. We applied PRS-CSx with both AA (or UKB AFR) and EUR GWAS summary statistics without using local ancestry information. Similarly as for pPRS, in real data analysis with evaluations internal in WHI, GWAS summary statistics were obtained from WHI WHIMS + EUR and WHI AA training individuals; in the UKB analysis, GWAS results were obtained from UKB EUR and UKB AFR training samples; in the cross-cohort evaluation analysis between WHI and UKB, GWAS results were derived from WHI WHIMS + EUR and UKB AFR. We used 1000 G AFR and EUR LD reference panels downloaded from the PRS-CSx GitHub. After calculating the posterior effect size of each variant, we adopted a two-fold cross-validation strategy to combine the EUR- and AFR- specific PRS. We split the testing dataset into two equal-size parts (part A and B), and used part A for deriving the weights to calculate the linearly combined PRS for each individual in part B. Then we switched the two parts and similarly calculated PRS for every individual in part A. In this way, one can argue that PRS-CSx is using more information than GAUDI and all comparisons were in situations unfavorable for GAUDI. When investigating the influence of the global shrinkage parameter tuning, we performed a small grid-search with 1, 1e-2, 1e-4 and 1e-6, as recommended by the authors. For all other experiments, we used the auto-selected parameter. Note that PRS-CSx was not included in the simulations because our simulated genotypes do not have real rsIDs, but PRS-CSx only allows real rsIDs.

For all the methods, PRS performance was assessed across 10 repeats for simulations and 5 folds for real data analysis. We used mean testing R^2^ between PRS and adjusted phenotypes for continuous traits, and the partial Nagelkerke’s R^2^ for binary traits^55^, as the evaluation metrics. For partial Nagelkerke’s R^2^, following the PRS-CSx work^18^, we calculated the difference of Nagelkerke’s R^2^ for model 1 (disease ~ covariates + PRS) and model 2 (disease ~ covariates) comparing each model to the null model using R package fmsb^55,56^.

### Evaluation of GAUDI using large-scale EUR ancillary GWAS for CRP and WBC in WHI AA

After observing highly encouraging advantages of GAUDI on CRP and WBC, we next evaluated whether GAUDI remains advantageous over PRS-CSx which could leverage large external GWAS summary statistics to construct PRS. We downloaded GWAS summary statistics from latest GWAS studies of CRP (*N* = 575,531)^26^ and WBC (*N* = 563,085)^27^. GAUDI still utilizes only the same admixed training individuals. PRSice was run with the large external EUR-based GWAS, with results from PRSice using only WHI AA training GWAS were also retained. PRS-CSx was performed using both WHI AA training GWAS and the downloaded external EUR-based GWAS. We also retained previous PRS-CSx results based on WHI AA training GWAS and WHI WHIMS + EUR GWAS to evaluate the benefits of including large-sample-size EUR GWAS.

### Trait screening in UKB: comparison with PRS-CSx under situations unfavorable for GAUDI

We performed comprehensive trait screening on 28 urine and serum biomarkers from UKB, to evaluate the potential benefit of GAUDI compared to PRS-CSx when PRS-CSx additionally leverages disproportionally powered EUR GWAS. Traits were selected using the same criteria as previously described^29^. We performed GWAS on the imputed data released from UKB (UK10K imputed) using REGENIE^48^, including variants with imputation INFO score >0.3 and MAC >20, separately for UKB EUR individuals (*N* ~ 430,000) and UKB AFR training individuals (*N* ~ 7000). We similarly adopted the outer-loop five-fold cross-validation strategy and performed GWAS separately on EUR and AFR individuals. For UKB AFR target individuals, we removed variants with INFO score <0.5 and MAF <0.1% when constructing PRS.

We compared GAUDI using only the ~7000 UKB AFR training individuals with PRS-CSx using GWAS from both the ~7000 UKB AFR and ~430,000 UKB EUR individuals. Methods were detailed in the PRS method application section above. We additionally added PRSice using GWAS from ~7000 UKB AFR training samples for comparison.

### Reporting summary

Further information on research design is available in the Nature Portfolio Reporting Summary linked to this article.

### Supplementary information

Supplementary Information

Description of Additional Supplementary Files

Supplementary Data 1

Supplementary Data 2

Reporting Summary

### Source data

Source Data

## Data Availability

WHI data are available through dbGaP Accession phs000200 or upon application to the WHI Coordinating Center (https://www.whi.org/) with approval required. UKB data are available upon request from UK Biobank (https://www.ukbiobank.ac.uk/) with approval required. 1000 Genomes data are publicly available from the consortium website (https://www.internationalgenome.org). TOPMed imputation reference panel can be accessed freely through the TOPMed imputation server (https://imputation.biodatacatalyst.nhlbi.nih.gov/#!). UKB GWAS summary statistics generated in this study are freely available to download at https://yunliweb.its.unc.edu/serum_biomarker/download.php. Large-scale European-based GWAS summary statistics for CRP and WBC are publicly available through https://www.ebi.ac.uk/gwas/studies/GCST90029070 (CRP) and http://www.mhi-humangenetics.org/en/resources (WBC). Pre-trained GAUDI models in the manuscript are publicly available to download at this FTP site: ftp://yunlianon:anon @rc-ns-ftp.its.unc.edu/GAUDI_models/. Source data are provided with this paper. All data supporting the findings described in this manuscript are available in the article and its Supplementary Information files, and from the corresponding author upon request. Source data are provided with this paper.

## Code availability

Codes used for analyses are available at https://github.com/quansun98/GAUDI/^57^.
