# Supplementary material for: Improving polygenic risk prediction in admixed populations by explicitly modeling ancestral-differential effects via GAUDI
Source: Nat Commun. 2024 Feb 3;15:1016. doi: 10.1038/s41467-024-45135-z (PMC10838303; doi:10.1038/s41467-024-45135-z)
Supplement: Supplementary file 1 — Supplementary Information [file 41467_2024_45135_MOESM1_ESM.pdf]

Improving polygenic risk prediction in admixed populations by explicitly modeling ancestral-differential effects via GAUDI

Supplementary Information

Supplementary Figures

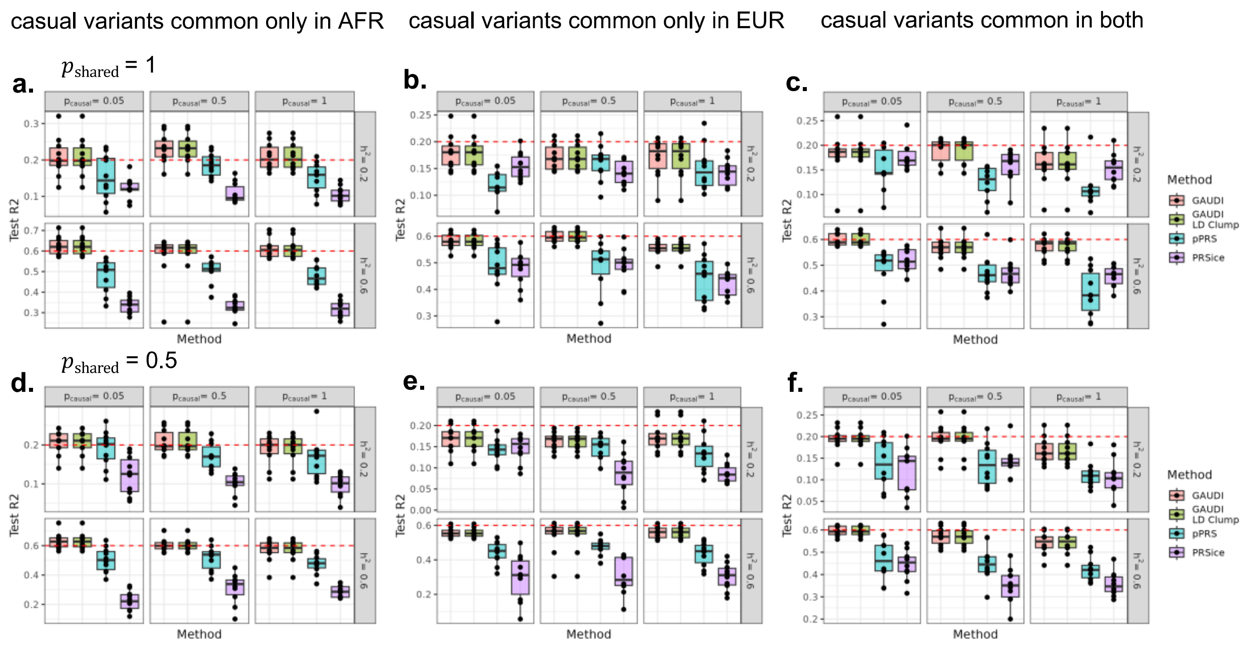

Supplementary Figure 1. GAUDI performance compared to PRSice and pPRS in small-scale simulation studies under different settings, with maximum LD R^2 between causal variants to be 0.2. (a)-(c). p_{shared} (proportion of variants with shared effects across ancestry groups) = 1: no ancestry-differential effects for all causal variants. **(d)-(e).** p_{shared} = 0.5: half of the causal variants have ancestry-differential effects. **(a)(d).** Causal variants are common only in AFR ancestry, specifically EUR-MAF < 5% and AFR-MAF >= 5%. **(b)(e).** Causal variants are common only in EUR ancestry, i.e., EUR-MAF >= 5% and AFR-MAF < 5%. **(c)(f).** Causal variants are common in both ancestries, i.e., EUR-MAF and AFR-MAF both >= 5%. Each experiment was repeated 10 times shown in the box plots. The minima, maxima and center represent the minimum, maximum and median test R^2 across the 10 repeats. The bounds of the boxes represent upper and lower quartiles, with whiskers represent 1.5 times of interquartile range. The maximum LD R^2 between causal variants were set to be 0.2 for all settings. The dashed red line denotes heritability. p_{causal}: proportion of causal variants out of all variants.

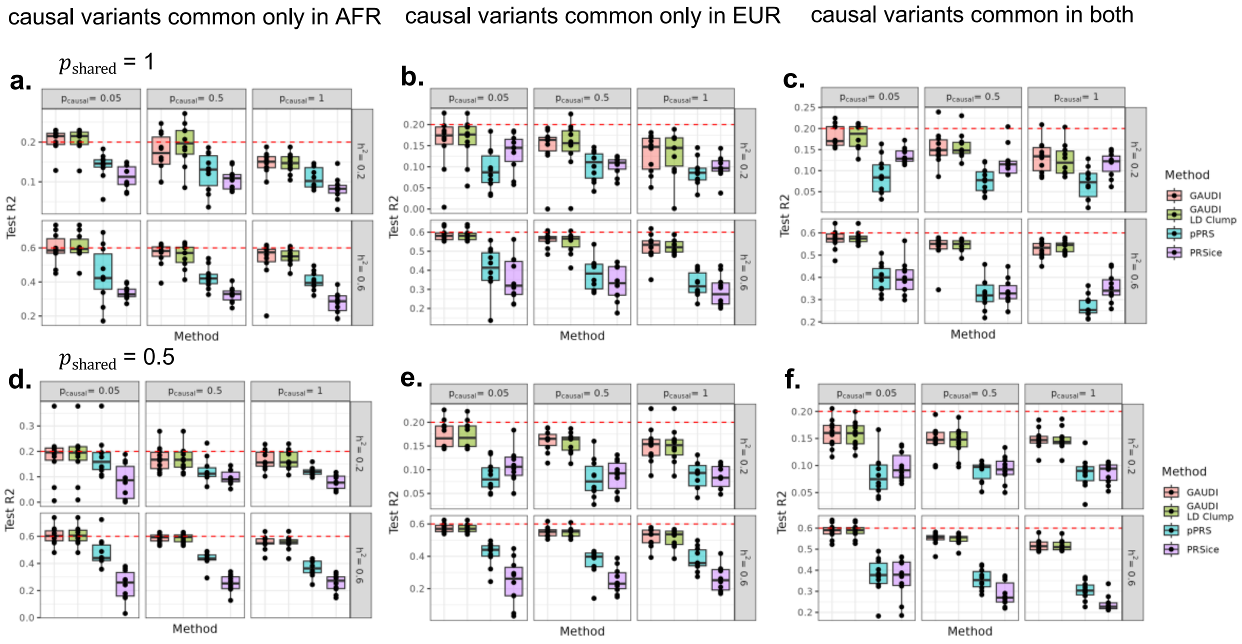

Supplementary Figure 2. GAUDI performance compared to PRSice and pPRS in small-scale simulation studies under different settings, with maximum LD R^2 between causal variants to be 0.5. (a)-(c). p_{shared} (proportion of variants with shared effects across ancestry groups) = 1: no ancestry-specific effects for all causal variants. **(d)-(e).** p_{shared} = 0.5: half of the causal variants have ancestry-specific effects. **(a)(d).** Causal variants are common only in AFR ancestry, specifically EUR-MAF < 5% and AFR-MAF >= 5%. **(b)(e).** Causal variants are common only in EUR ancestry, i.e., EUR-MAF >= 5% and AFR-MAF < 5%. **(c)(f).** Causal variants are common in both ancestries, i.e., EUR-MAF and AFR-MAF both >= 5%. Each experiment was repeated 10 times shown in the box plots. The minima, maxima and center represent the minimum, maximum and median test R^2 across the 10 repeats. The bounds of the boxes represent upper and lower quartiles, with whiskers represent 1.5 times of interquartile range. The maximum LD R2 between causal variants were set to be 0.5 for all the settings. The dashed red line denotes heritability. p_{causal}: proportion of causal variants out of all variants.

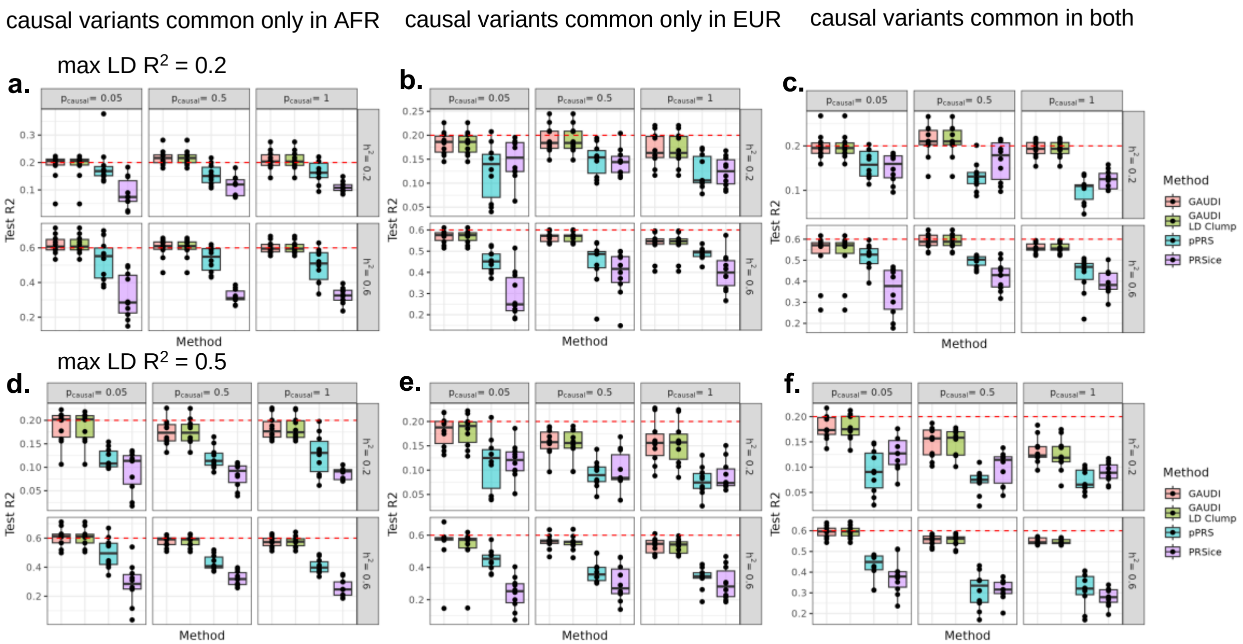

Supplementary Figure 3. GAUDI performance compared to PRSice and pPRS in small-scale simulation studies under different settings, with the proportion of ancestry-specific causal variants being 20%. (a)-(c). The maximum LD R^2 between causal variants were set to be 0.2. **(d)-(e).** The maximum LD R^2 between causal variants were set to be 0.5. **(a)(d).** Causal variants are common only in AFR ancestry, specifically EUR-MAF < 5% and AFR-MAF >= 5%. **(b)(e).** Causal variants are common only in EUR ancestry, i.e., EUR-MAF >= 5% and AFR-MAF < 5%. **(c)(f).** Causal variants are common in both ancestries, i.e., EUR-MAF and AFR-MAF both >= 5%. Each experiment was repeated 10 times shown in the box plots. The minima, maxima and center represent the minimum, maximum and median test R^2 across the 10 repeats. The bounds of the boxes represent upper and lower quartiles, with whiskers represent 1.5 times of interquartile range. The proportion of ancestry-specific causal variants was set to be 20% for all the settings. The dashed red line denotes heritability.

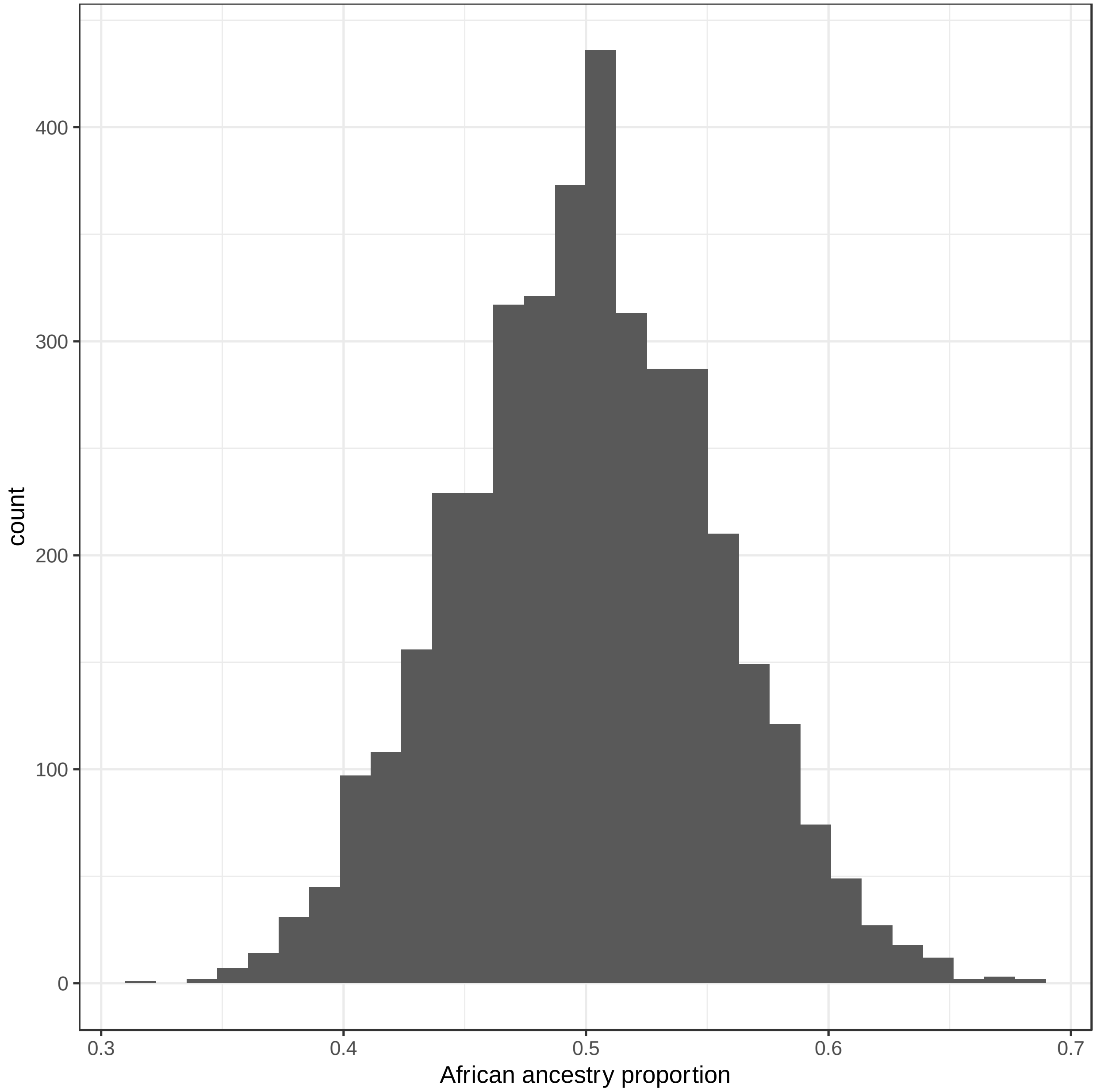

Supplementary Figure 4. Histogram of average African ancestry proportion for simulated AA individuals (n=3920). We assumed the proportion of AFR components follows N(0.5, 0.003) distribution ignoring negative values.

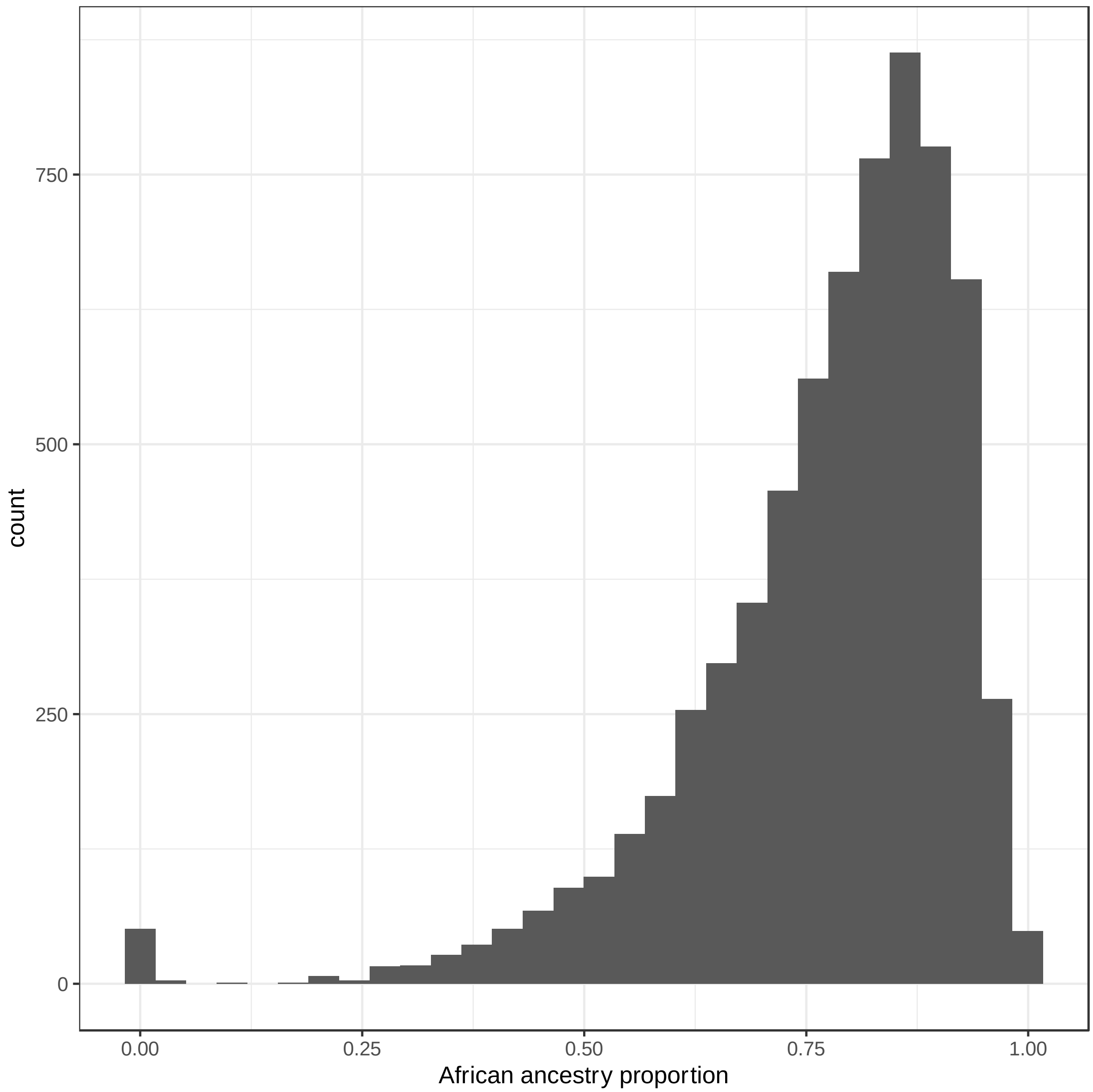

Supplementary Figure 5. Histogram of average African ancestry proportion for WHI AA individuals (n=6734). We inferred the 2-way local ancestry for WHI AA and summarized at genome-wide level with 22 chromosomes considered.

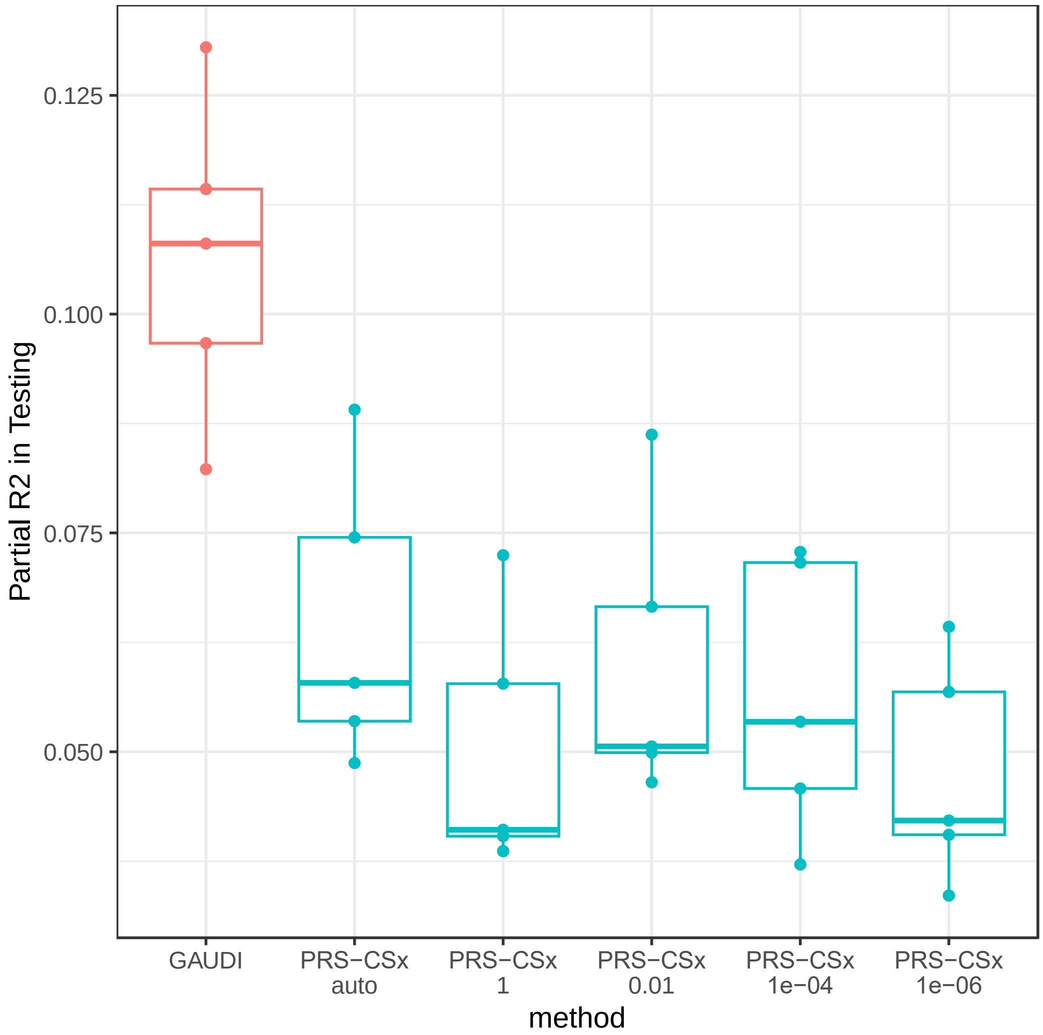

Supplementary Figure 6. Performance of PRS-CSx under different values of the global shrinkage parameter in WHI AA internal evaluations for WBC. We show that the advantage of GAUDI over PRS-CSx was not due to not carefully tuning the parameter for PRS-CSx. The PRS-CSx-auto, which we used in all the analyses, performed the best across the small grid search. For each boxplot, the minima, maxima and center represent the minimum, maximum and median test R^2 across the 5 repeats. The bounds of the boxes represent upper and lower quartiles, with whiskers represent 1.5 times of the interquartile range.

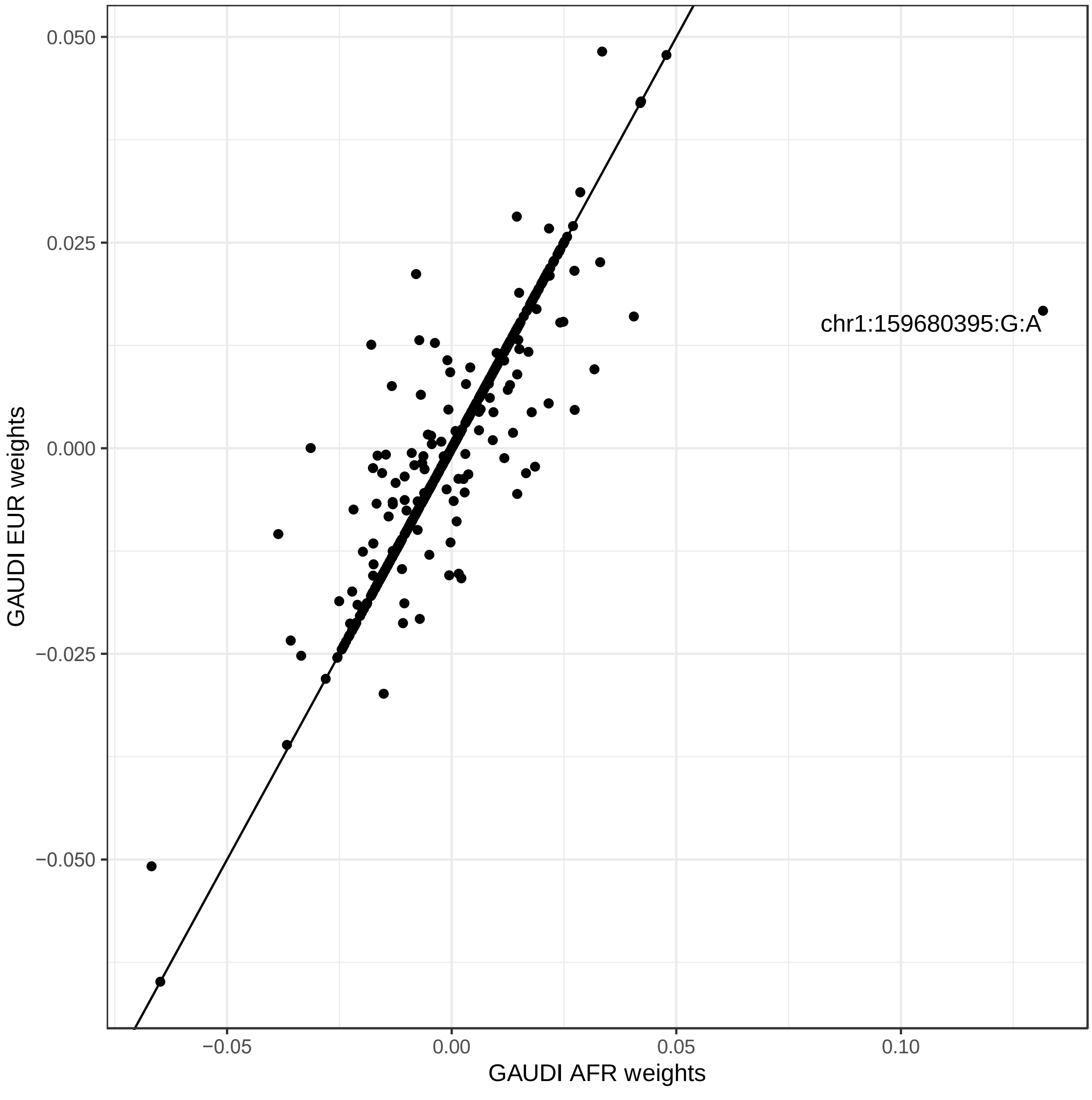

Supplementary Figure 7. Comparison of GAUDI EUR weights and AFR weights for CRP. We identified one variant with significant differential effects between EUR and AFR. This variant has extremely low MAF in EUR (MAF = 0.05%) but is common in AFR (MAF = 19.8%).

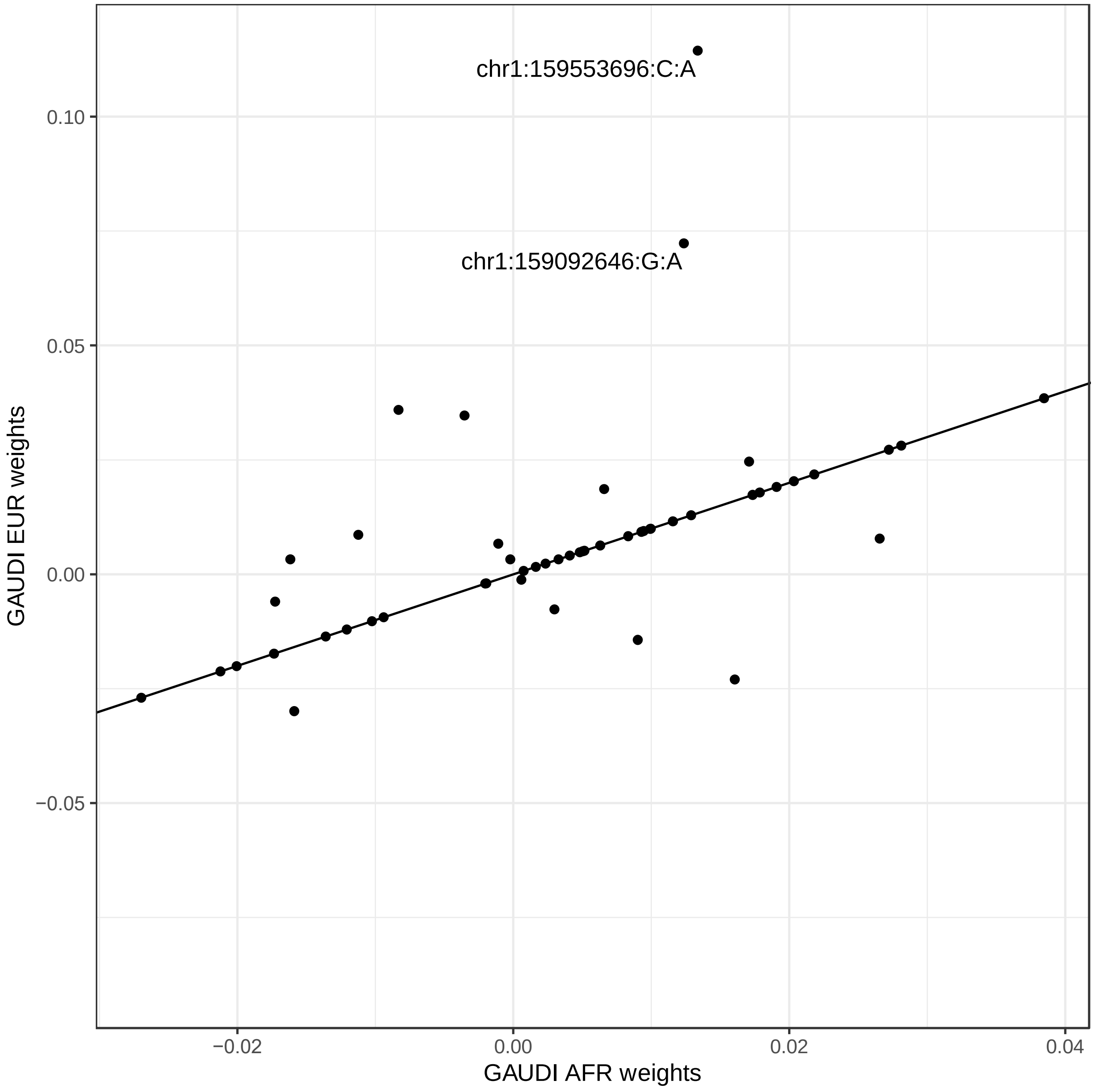

Supplementary Figure 8. Comparison of GAUDI EUR weights and AFR weights for WBC. We identified two variants with significant differential effects between EUR and AFR. Of note that chr1:159092646:G:A is in moderately high LD (R^2 0.6) with the Duffy null variant in 1000G Africans.

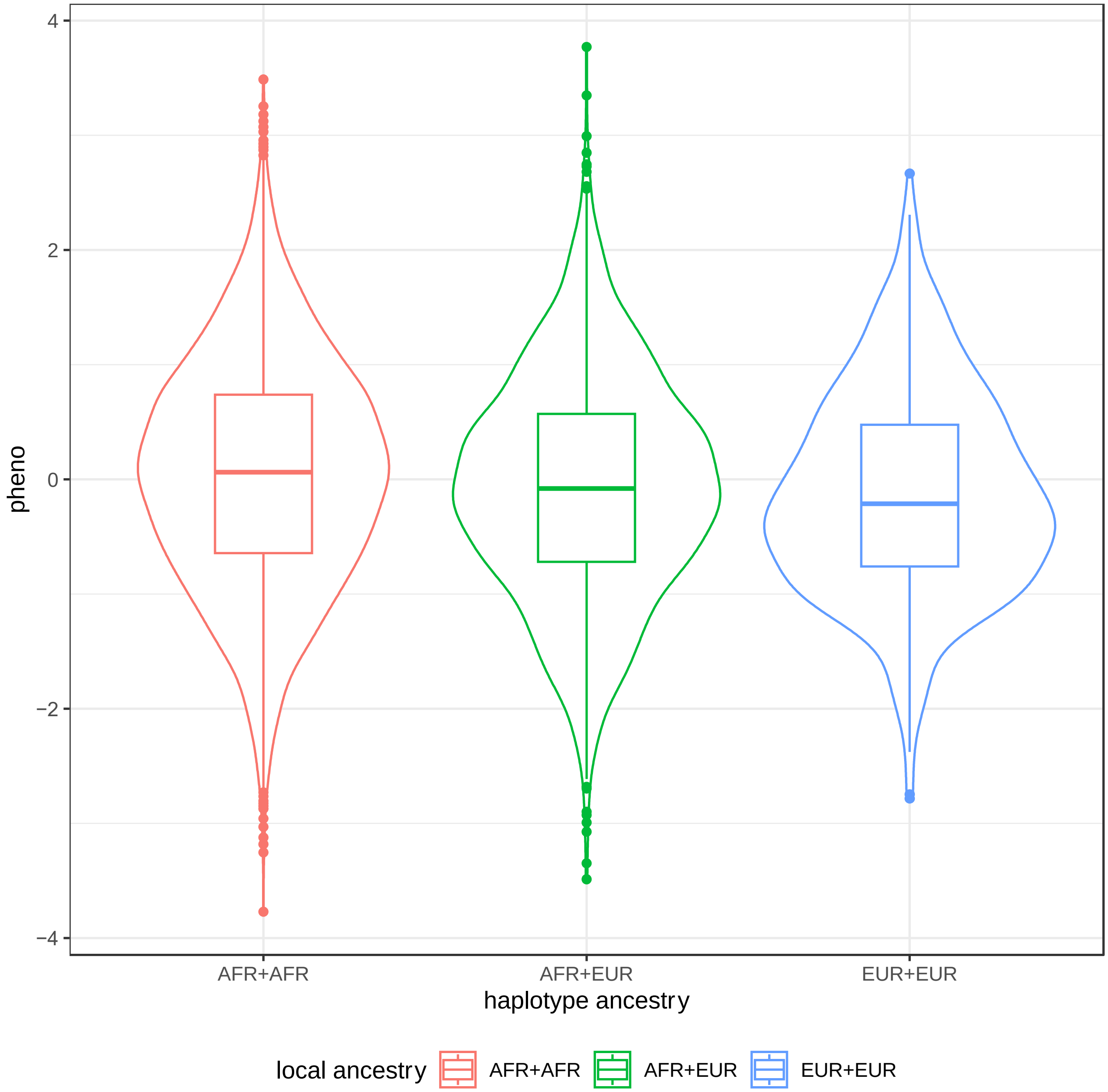

Supplementary Figure 9. Local ancestry effects of the variant outlier in the CRP example. The y-axis shows the adjusted CRP values (after adjusting for covariates and performing inverse normal transformation). The x-axis classifies individuals according to their inferred local ancestry at this locus. For example, AFR+AFR means individuals with both alleles from African ancestry at this variant. Color represents different local ancestry information.

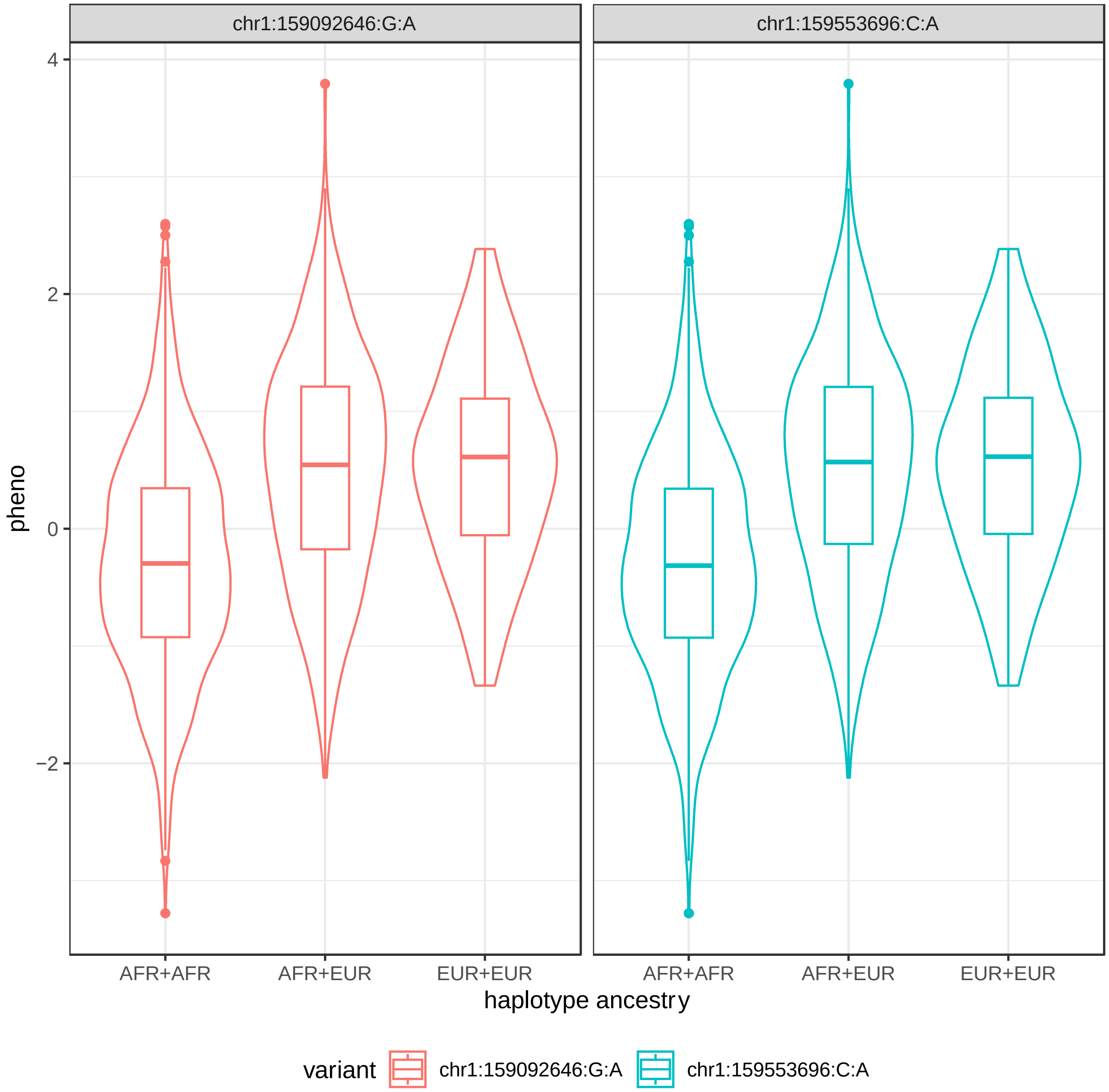

Supplementary Figure 10. Local ancestry effects of the two variant outliers in the WBC example. The y-axis shows the adjusted WBC values (after adjusting for covariates and performing inverse normal transformation). The x-axis classifies individuals according to their inferred local ancestry at each variant. For example, AFR+AFR means individuals with both alleles from African ancestry at this variant. Color represents the two genetic variants.

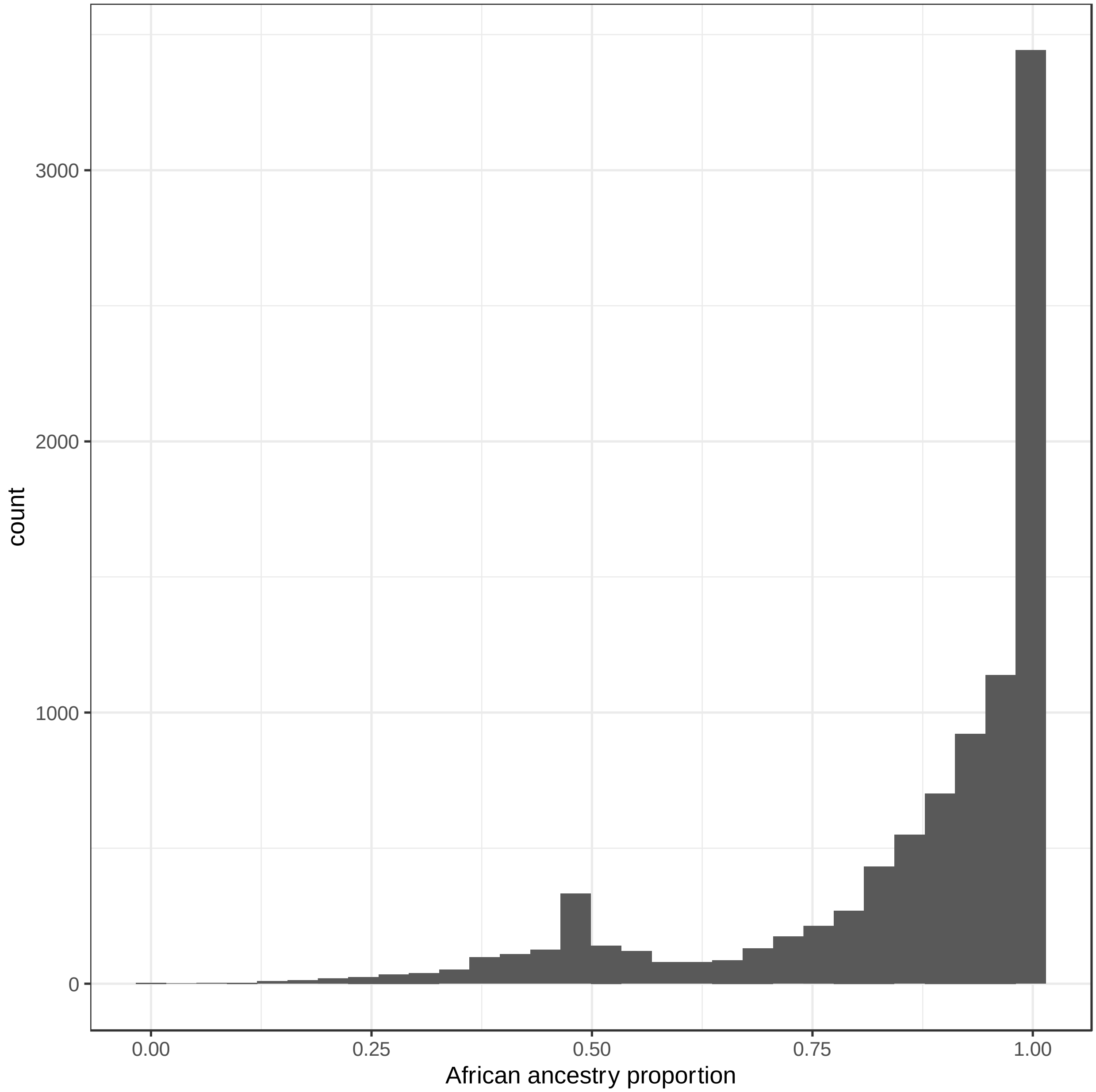

Supplementary Figure 11. Histogram of average African ancestry proportion for UKB AFR individuals (n=9354). We inferred the 2-way local ancestry for UKB participants with African components and summarized at genome-wide level with 22 chromosomes considered.

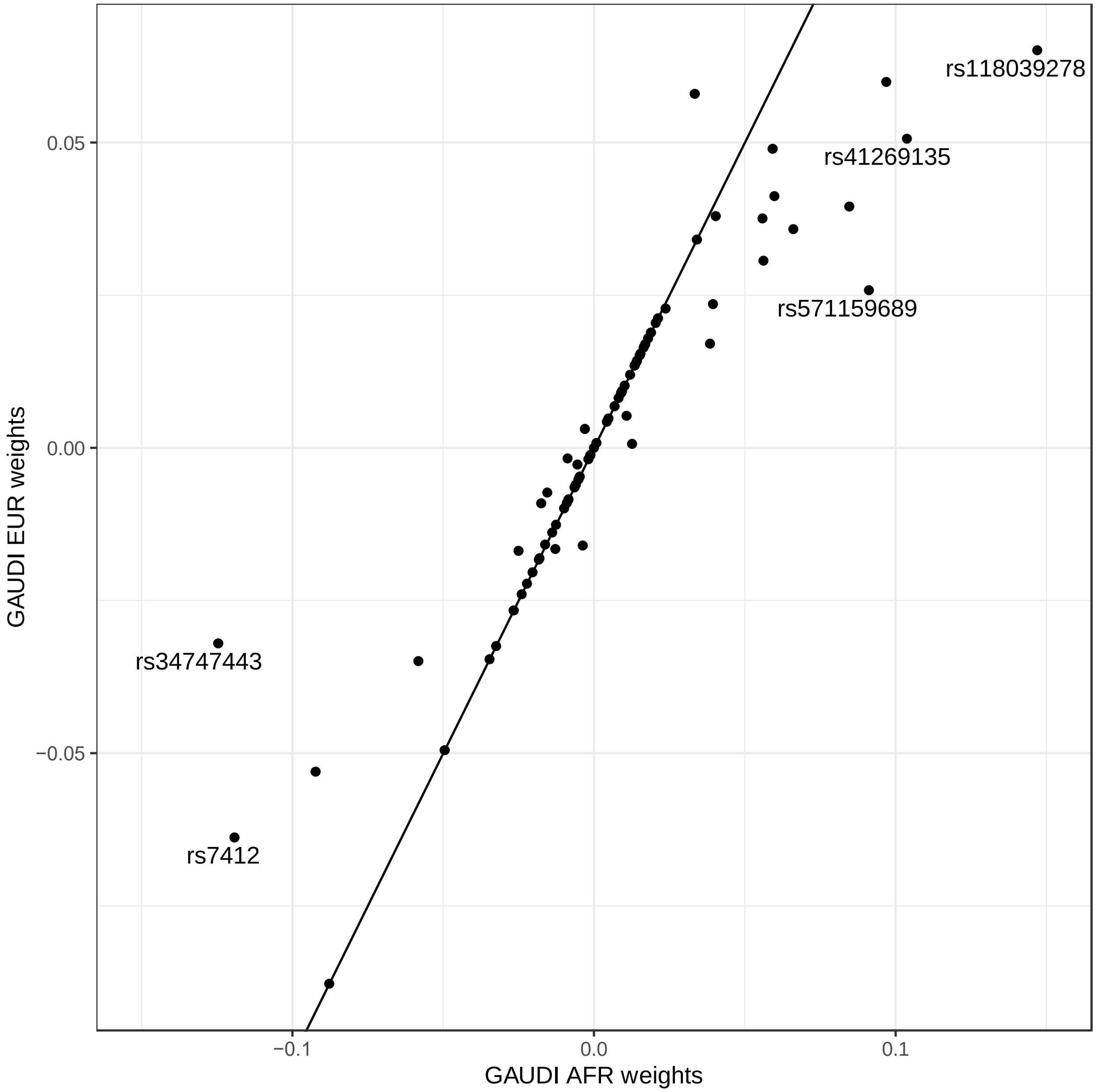

Supplementary Figure 12. Comparison of GAUDI EUR weights and AFR weights for lipoprotein A in UKB serum and urine biomarker screening. We identified several variants with significant differential effects between EUR and AFR.

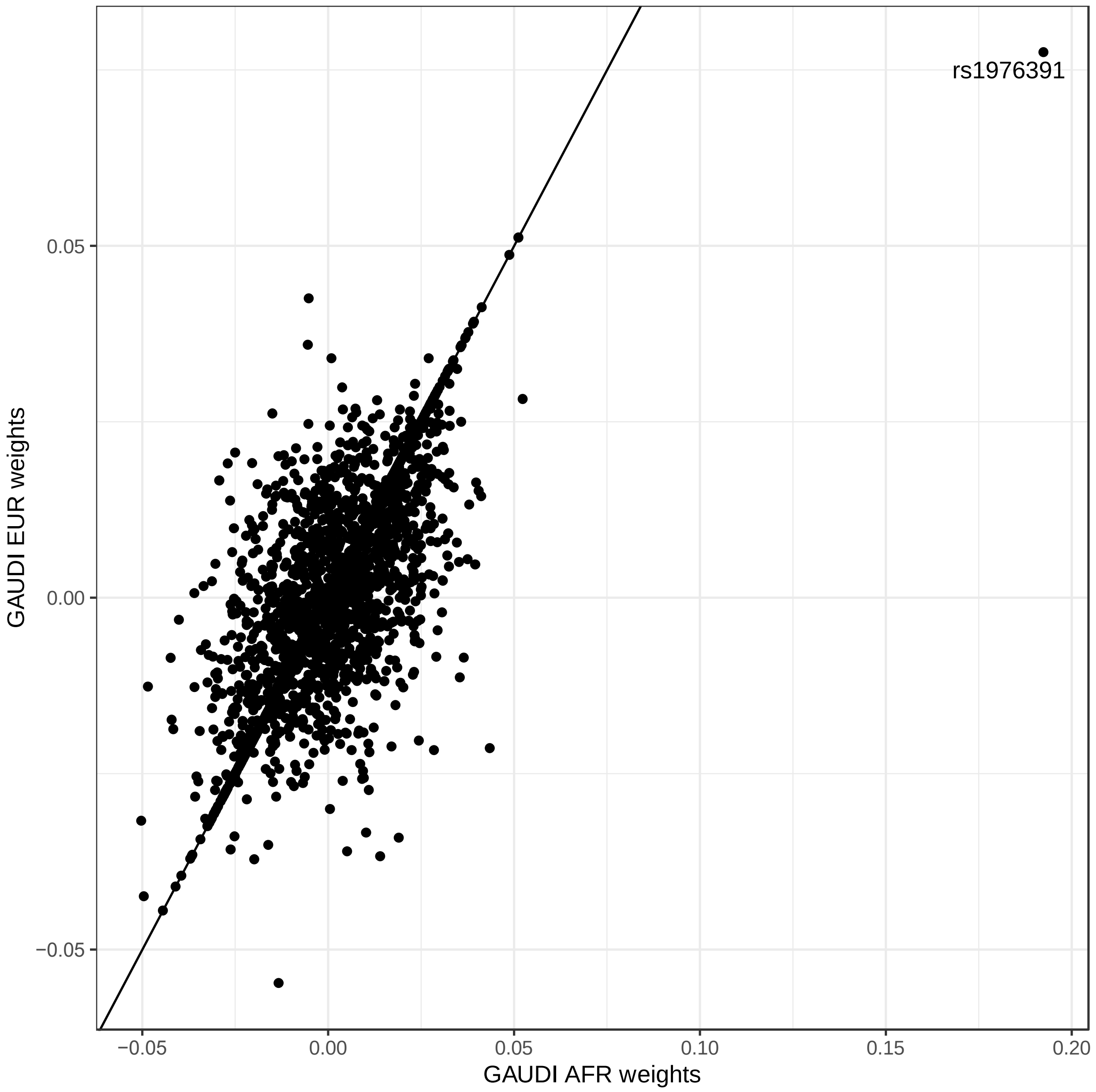

Supplementary Figure 13. Comparison of GAUDI EUR weights and AFR weights for direct bilirubin in UKB serum and urine biomarker screening. We identified one obvious outlier variant, rs1976391, with significant differential effects between EUR and AFR.

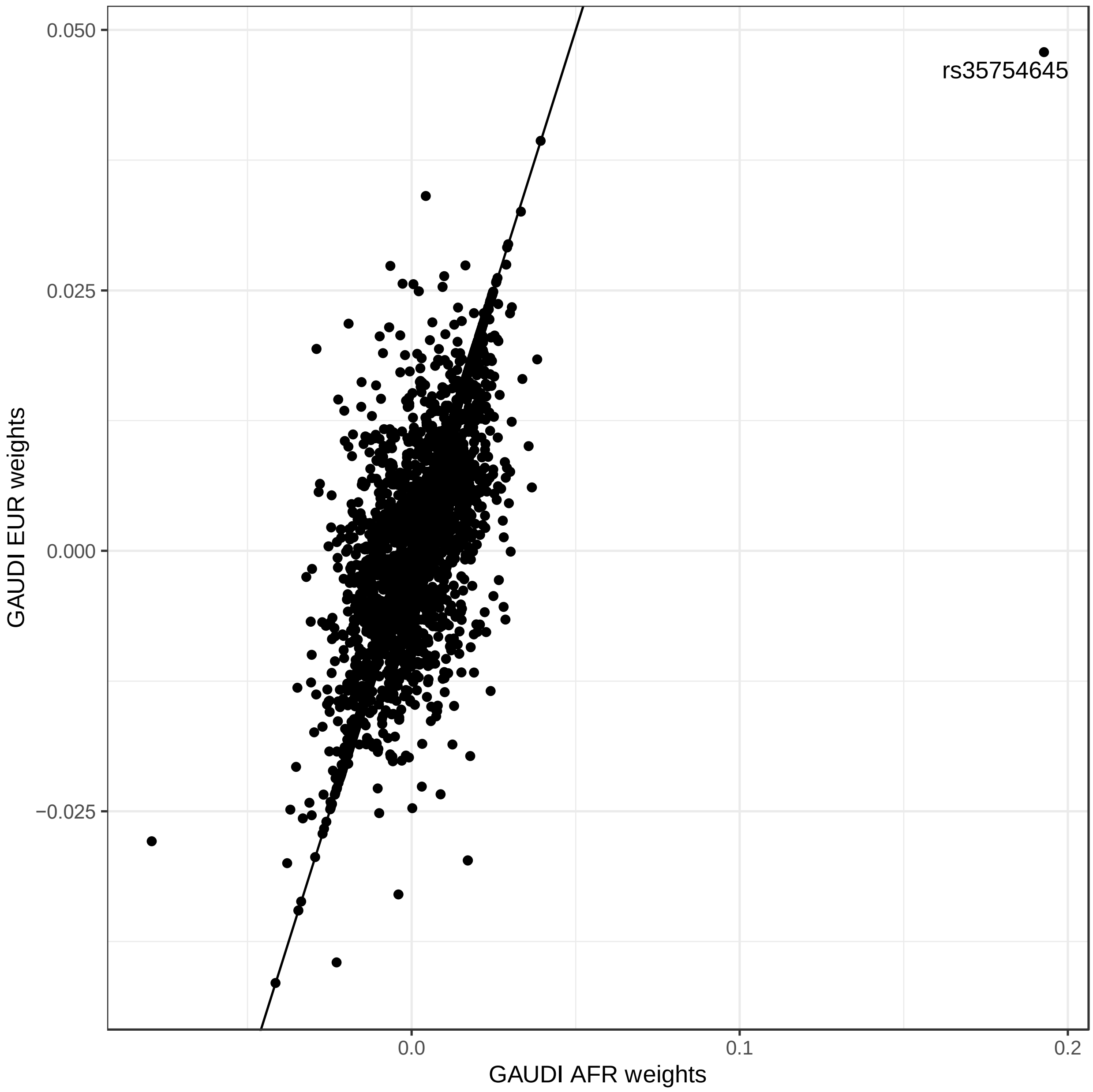

Supplementary Figure 14. Comparison of GAUDI EUR weights and AFR weights for total bilirubin in UKB serum and urine biomarker screening. We identified one obvious outlier variant, rs35754645, with significant differential effects between EUR and AFR.

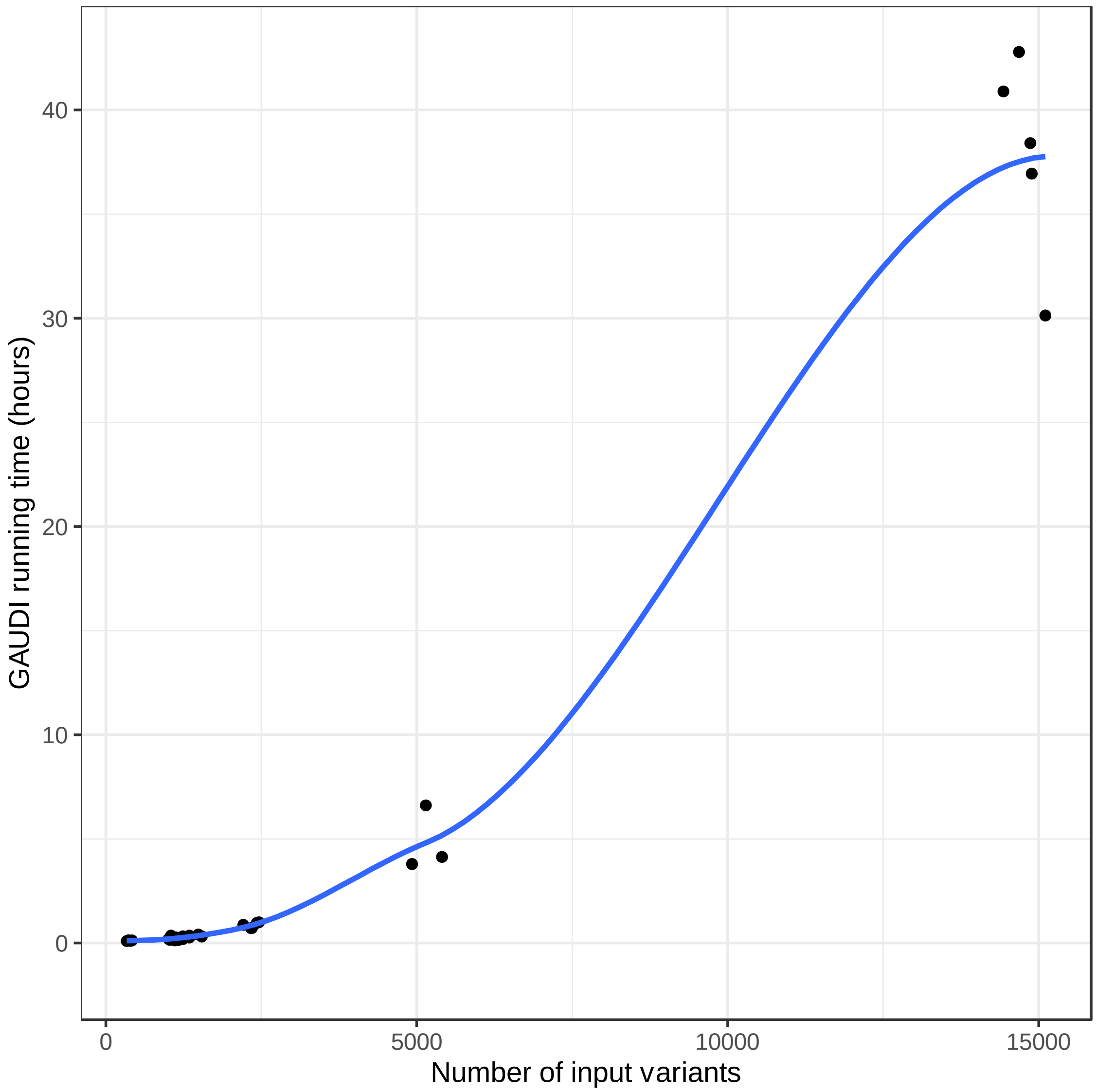

Supplementary Figure 15. Assessment of computational time of GAUDI with different number of input variants in real data analyses. The computational time depends heavily on the number of input variants, with exponential rate increase. We note that the computational bottleneck is the largest limitation of GAUDI.

Supplementary Note

Small-scale simulation results

We first compared GAUDI with the clumping and thresholding method implemented in PRSice [1] and the previously proposed partial PRS (pPRS) [2] method, under the scenario of no ancestry-specific effects (p_{shared} = 1, **Methods**). While 100% of effects being shared across ancestry is an over-simplification, recent work has shown that there is almost always a positive correlation between effect sizes across global populations for most variants associated with complex traits [3]. We ran PRSice, pPRS, GAUDI with and without LD clumping for comparison (**Methods**). We used COSI [4] to simulate 102,572 genetic variants for 3,500 AA individuals assuming 80% African (AFR) and 20% European (EUR) admixture, and another independent samples of 2,500 EUR and 2,500 AFR individuals serving as references. We considered three different genetic settings of the causal variants in terms of their minor allele frequency (MAF) across ancestries: variants with EUR-MAF and AFR-MAF both >= 5% (causal variants common in both ancestries), variants with EUR-MAF >= 5% and AFR-MAF < 5% (casual variants common only in EUR), and variants with EUR-MAF < 5% and AFR-MAF >= 5% (causal variants common only in AFR). For each of three MAF settings, we varied the proportion of causal variants to be 1, 0.5, 0.05 to represent different polygenicity situations, and the proportion of variation explained by genetic variations (i.e., heritability) to be 0.2 or 0.6. In addition, we also varied the maximum LD R^2 among causal variants to be 0.2 or 0.5.

Comparing across different polygenicity and heritability scenarios, GAUDI achieved best performance across the entire spectrum assessed, demonstrating most pronounced performance gains in settings with higher heritability and denser genetic architecture. In addition, the R^2 attained by GAUDI in the testing dataset is nearly equal to heritability in almost all simulated phenotypes, demonstrating the power of GAUDI by borrowing information from haplotype segments in one ancestry to better estimate the effects in another ancestry.

We then simulated phenotypes where 50% of causal variants have ancestry-specific effects and the remaining 50% have effect sizes shared across the two ancestral populations (**Methods**). Similarly, we considered multiple genetic architectures by varying causal variants' cross-ancestry MAFs, heritability, polygenicity and maximum LD R^2. Our results were largely consistent with those from the above simulations (**Supplementary Fig. 1-2 d-f, Supplementary Table 2**). The improvement of GAUDI over competing methods is even more pronounced in some scenarios with the introduction of ancestry-specific effects (**Supplementary Fig. 1**). We also note the variability of GAUDI is slightly reduced compared to the previous setting where no ancestry-specific effects were allowed. These results further underscore the advantage of GAUDI by allowing and jointly modeling ancestry-specific effects. We also simulated phenotypes where the proportion of ancestry-specific causal variants is 20%, and the results are highly consistent (**Supplementary Figure 3**), suggesting GAUDI is robust to a variety of genetic architectures. Furthermore, GAUDI with LD clumping performs almost identically well as GAUDI without LD clumping, indicating GAUDI is also robust to the inclusion of correlated variants in the PRS construction process.

Outliers in the WBC experiments

We found two outliers in the WBC experiments, chr1:159092646:G:A (rs2518564) and chr1:159553696:C:A (rs2084257). Both variants demonstrate higher EUR weights than AFR weights in GAUDI (**Supplementary Fig. 8**). We note that the weights plotted are from GAUDI rather than GWAS. Our GAUDI weights are re-estimated in a joint manner, jointly for ancestry-specific weights across all variants, which are different from GWAS effect sizes that are estimated in a marginal manner one variant at a time.

Going back to the two outliers, we first note that their GWAS effect sizes and p-values are not particularly large or highly significant, with effect sizes 6.3e-3 and 0.014, p-values 0.018 and 4.9e-5 for chr1:159092646:G:A and chr1:159553696:C:A, respectively, in the 500k EUR WBC GWAS [5]. Future studies with finer imputation reference panel including the Duffy null variant in larger sample sizes of African continental or African Americans are warranted for a more comprehensive understanding. More interestingly, the local ancestry at the two variants seems to be associated with the phenotype (**Supplementary Fig. 9**). Specifically, individuals with at least one copy of European local ancestry alleles (middle and right violins in the plots) tend to have higher values of white blood cell counts than those with both alleles of African local ancestry (leftmost violins). In this sense, the weights from GAUDI shall be interpreted differently from how we typically interpret variant effect sizes, because the weights here change the predicted values of white blood cell counts on top of the linear combinations of other variant and local ancestry combinations. The seemingly smaller AFR effect sizes are consistent with the observed (representing working truth) lower values of white blood cell counts among individuals carrying two copies of African alleles.

Supplementary References

1. Choi, S. W. & O'Reilly, P. F. PRSice-2: Polygenic Risk Score software for biobank-scale data. *Gigascience* **8**, (2019).

2. Marnetto, D. *et al.* Ancestry deconvolution and partial polygenic score can improve susceptibility predictions in recently admixed individuals. *Nat. Commun.* **11**, 1628 (2020).

3. Veturi, Y. *et al.* Modeling heterogeneity in the genetic architecture of ethnically diverse groups using random effect interaction models. *Genetics* **211**, 1395–1407 (2019).

4. Schaffner, S. F. *et al.* Calibrating a coalescent simulation of human genome sequence variation. *Genome Res.* **15**, 1576–1583 (2005).

5. Vuckovic, D. *et al.* The polygenic and monogenic basis of blood traits and diseases. *Cell* **182**, 1214-1231.e11 (2020).
