# Supplementary material for: Improving polygenic risk prediction in admixed populations by explicitly modeling ancestral-differential effects via GAUDI
Source: Nat Commun. 2024 Feb 3;15:1016. doi: 10.1038/s41467-024-45135-z (PMC10838303; doi:10.1038/s41467-024-45135-z)
Supplement: Supplementary file 2 — Description of Additional Supplementary Files [file 41467_2024_45135_MOESM2_ESM.pdf]

Description of Additional Supplementary Files

File Name: Supplementary Data 1
Description: Detailed simulation results under no ancestry-specific effects. Comparison between GAUDI without LD clumping, GAUDI with LD clumping, PRSice and pPRS. We varied heritability to be 0.2 and 0.6, proportion of causal variants (p_causal) to be 0.05, 0.5 and 1, the maximum LD R2 between causal SNPs to be 0.2 and 0.5, and evaluated three different causal variants MAF settings. Each experiment was repeated for 10 times.

File Name: Supplementary Data 2
Description: Performance comparison of GAUDI to PRSice and PRS-CSx in the UKB trait screening. GAUDI and PRSice leverages the same training individuals (UKB AFR, N ~ 7000), while PRS-CSx leverages GWAS summary statistics from both UKB AFR (N ~ 7000) and UKB EUR (N ~ 430,000). All the numbers are mean R2 across five outer-loop cross validations.
