# Supplementary material for: Improving polygenic risk prediction in admixed populations by explicitly modeling ancestral-differential effects via GAUDI
Source: Nat Commun. 2024 Feb 3;15:1016. doi: 10.1038/s41467-024-45135-z (PMC10838303; doi:10.1038/s41467-024-45135-z)
Supplement: Supplementary file 5 — Reporting Summary [file 41467_2024_45135_MOESM5_ESM.pdf]

nature portfolio

Corresponding author(s): Yun Li
Last updated by author(s): Jan 3, 2024

Reporting Summary

Nature Portfolio wishes to improve the reproducibility of the work that we publish. This form provides structure for consistency and transparency in reporting. For further information on Nature Portfolio policies, see our Editorial Policies and the Editorial Policy Checklist.

Statistics

For all statistical analyses, confirm that the following items are present in the figure legend, table legend, main text, or Methods section.

n/a	Confirmed	
☐	☒	The exact sample size (*n*) for each experimental group/condition, given as a discrete number and unit of measurement
☒	☐	A statement on whether measurements were taken from distinct samples or whether the same sample was measured repeatedly
☐	☒	The statistical test(s) used AND whether they are one- or two-sided<br>*Only common tests should be described solely by name; describe more complex techniques in the Methods section.*
☐	☒	A description of all covariates tested
☐	☒	A description of any assumptions or corrections, such as tests of normality and adjustment for multiple comparisons
☐	☒	A full description of the statistical parameters including central tendency (e.g. means) or other basic estimates (e.g. regression coefficient) AND variation (e.g. standard deviation) or associated estimates of uncertainty (e.g. confidence intervals)
☐	☒	For null hypothesis testing, the test statistic (e.g. *F*, *t*, *r*) with confidence intervals, effect sizes, degrees of freedom and *P* value noted<br>*Give P values as exact values whenever suitable.*
☒	☐	For Bayesian analysis, information on the choice of priors and Markov chain Monte Carlo settings
☒	☐	For hierarchical and complex designs, identification of the appropriate level for tests and full reporting of outcomes
☐	☒	Estimates of effect sizes (e.g. Cohen's *d*, Pearson's *r*), indicating how they were calculated

Our web collection on statistics for biologists contains articles on many of the points above.

Software and code

Policy information about availability of computer code

Data collection	N/A
Data analysis	All software and code used in the text are now reported in the text along with their version numbers. The software and codes for GAUDI are deposited on GitHub page https://github.com/quansun98/GAUDI/. In addition, we used the following software: R v4.1.0, python v3.7.9, COSI v1.2.1, bcftools v1.16, plink v1.90 beta, plink v2.00 alpha, PRSice v2.3.3, RFMix v2, REGENIE v3.1.3, Eagle v2.4, minimac4 v1.0.2, and PRS-CSx with most recent revision on July 29, 2021.

For manuscripts utilizing custom algorithms or software that are central to the research but not yet described in published literature, software must be made available to editors and reviewers. We strongly encourage code deposition in a community repository (e.g. GitHub). See the Nature Portfolio guidelines for submitting code & software for further information.

Data

Policy information about availability of data

All manuscripts must include a data availability statement. This statement should provide the following information, where applicable:

- Accession codes, unique identifiers, or web links for publicly available datasets
- A description of any restrictions on data availability
- For clinical datasets or third party data, please ensure that the statement adheres to our policy

WHI data are available through dbGaP Accession phs000200 or upon application to the WHI Coordinating Center (https://www.whi.org/) with approval required.

UKB data are available upon request from UK Biobank (https://www.ukbiobank.ac.uk/) with approval required. 1000 Genomes data are publicly available from the consortium website (https://www.internationalgenome.org). TOPMed imputation reference panel can be accessed freely through the TOPMed imputation server (https://imputation.biodatacatalyst.nhlbi.nih.gov/#!). UKB GWAS summary statistics generated in this study is freely available to download at https://yunliweb.its.unc.edu/serum_biomarker/download.php. Large-scale European-based GWAS summary statistics for CRP and WBC are publicly available through https://www.ebi.ac.uk/gwas/studies/GCST90029070 (CRP) and http://www.mhi-humangenetics.org/en/resources (WBC). Pre-trained GAUDI models in the manuscript are publicly available to download at this FTP site: ftp://yunlianon:anon@rc-ns-ftp.its.unc.edu/GAUDI_models/. Source data are provided with this paper. All data supporting the findings described in this manuscript are available in the article and its Supplementary Information files, and from the corresponding author upon request.

Research involving human participants, their data, or biological material

Policy information about studies with human participants or human data. See also policy information about sex, gender (identity/presentation), and sexual orientation and race, ethnicity and racism.

Reporting on sex and gender	This study does not involve study participants recruitment, neither collection of sex and/or gender information. In our analyses, self-reported sex was included as a covariate to account for its potential effects.
Reporting on race, ethnicity, or other socially relevant groupings	This study does not involve any race, ethnicity or socially relevant information.
Population characteristics	WHI PAGE GWAS participants were genotyped by the Population Architecture using Genomics and Epidemiology (PAGE) study using the Multi-Ethnic Genotyping Array (MEGA) array. UKB participants were genotyped by UKB with information available at https://biobank.ctsu.ox.ac.uk/crystal/label.cgi?id=263.
Recruitment	This study does not involve study participants recruitment.
Ethics oversight	N/A

Note that full information on the approval of the study protocol must also be provided in the manuscript.

Field-specific reporting

Please select the one below that is the best fit for your research. If you are not sure, read the appropriate sections before making your selection.

☒ Life sciences ☐ Behavioural & social sciences ☐ Ecological, evolutionary & environmental sciences

For a reference copy of the document with all sections, see nature.com/documents/nr-reporting-summary-flat.pdf

Life sciences study design

All studies must disclose on these points even when the disclosure is negative.

Sample size	No statistical methods were used to predetermine sample size. We used the maximum of samples with both genotype and phenotype information available.
Data exclusions	No data were excluded from the analyses.
Replication	All attempts at replication were successful and are described in the text. We performed five independent repeats for each computational experiment, which is not affected by time or location of the replications.
Randomization	There was no randomization involved. We included covariates to account for potential confounding effects, including age, sex, genotyping array, recruitment center.
Blinding	Blinding is not relevant to our study because we do not apply any differential treatment, intervention, or perturbation to our samples.

Reporting for specific materials, systems and methods

We require information from authors about some types of materials, experimental systems and methods used in many studies. Here, indicate whether each material, system or method listed is relevant to your study. If you are not sure if a list item applies to your research, read the appropriate section before selecting a response.

Materials & experimental systems

n/a	Involved in the study
☒	☐ Antibodies
☒	☐ Eukaryotic cell lines
☒	☐ Palaeontology and archaeology
☒	☐ Animals and other organisms
☒	☐ Clinical data
☒	☐ Dual use research of concern
☒	☐ Plants

Methods

n/a	Involved in the study
☒	☐ ChIP-seq
☒	☐ Flow cytometry
☒	☐ MRI-based neuroimaging

Plants

Seed stocks

Report on the source of all seed stocks or other plant material used. If applicable, state the seed stock centre and catalogue number. If plant specimens were collected from the field, describe the collection location, date and sampling procedures.

Novel plant genotypes

Describe the methods by which all novel plant genotypes were produced. This includes those generated by transgenic approaches, gene editing, chemical/radiation-based mutagenesis and hybridization. For transgenic lines, describe the transformation method, the number of independent lines analyzed and the generation upon which experiments were performed. For gene-edited lines, describe the editor used, the endogenous sequence targeted for editing, the targeting guide RNA sequence (if applicable) and how the editor was applied.

Authentication

Describe any authentication procedures for each seed stock used or novel genotype generated. Describe any experiments used to assess the effect of a mutation and, where applicable, how potential secondary effects (e.g. second site T-DNA insertions, mosiacism, off-target gene editing) were examined.
